# Supplementary material for: Human occupations at the Alpysbaev Cave (western Tian Shan): Bioarchaeological insights from the Iron Age burial cluster
Source: PLoS One. 2025 Dec 11;20(12):e0333708. doi: 10.1371/journal.pone.0333708 (PMC12697947; doi:10.1371/journal.pone.0333708)
Supplement: S1 File — (DOCX) [file pone.0333708.s001.docx]

Supplementary information for

Human occupations at the Alpysbaev Cave (western Tian Shan): Bioarchaeological insights from the Iron Age burial cluster

Abay Namen^1,2*^, Saine C. Hernandez Burgos^3*^, Aristeidis Varis^2,4^, Emily Coco^3^, Rachel Kalisher^3^, Emily Gaul^5^, Guido Alberto Gnecchi-Ruscone^6^, Maria A. Spyrou^5,6^, Cosimo Posth^5,6^, Susanne Lindauer^7^, David Naumann^4^, Scott A. Williams^3^, Zhaken Taimagambetov^8^, Radu Iovita^2,3^

1 Department of Sociology and Anthropology, School of Sciences and Humanities, Nazarbayev University, Astana 010000, Kazakhstan

2 Early Prehistory and Quaternary Ecology, Department of Geosciences,University of Tübingen, Tübingen 72074, Germany

3 Center for the Study of Human Origins, Department of Anthropology, New York University, New York 10003, USA

4 Institute for Archaeological Sciences, University of Tübingen, Tübingen 72074, Germany

5 Archaeo- and Palaeogenetics, Institute for Archaeological Sciences, Department of Geosciences, University of Tübingen, Tübingen 72074, Germany

6 Senckenberg Centre for Human Evolution and Palaeoenvironment at the University of Tübingen, Tübingen 72074, Germany

7 Curt-Engelhorn-Centre for Archaeometry, Mannheim 68159, Germany

8 National Museum of the Republic of Kazakhstan, Astana 010000, Kazakhstan

* contributed equally

This file includes:

Supplementary Text 1

Figures S1-S38

Table S1

***Supplementary Text 1***

**Description of all identified human skeletal remains**

This supplementary text provides further photos and information about each of the human skeletal elements mentioned in this study. A summary of the element counts is shown in Table S1. Photos of elements were taken by SCHB and are shown in Figs S1-S37.

**Table S1.** Inventory of types of bone and number of bones present

| Type of Element | Number of Individual Elements |
| --- | --- |
| Tooth | 6 |
| Vertebra | 6 |
| Rib | 11 |
| Clavicle | 3 |
| Scapula | 3 |
| Humerus | 1 |
| Os coxa | 2 |
| Phalanx (hand and foot) | 7 |
| Tarsal | 1 |
| Other | 1 |
| total | 41 |

## **VERTEBRAE & RIBS**

### *TPIT1-523:* A well-preserved adult, second cervical vertebra (C2, Axis) (Fig. S1). Only the left transverse process and a portion of the left part of the bifurcated spinous process are missing postmortem. A small osteophyte is present on the right portion of the bifurcated spinous process.


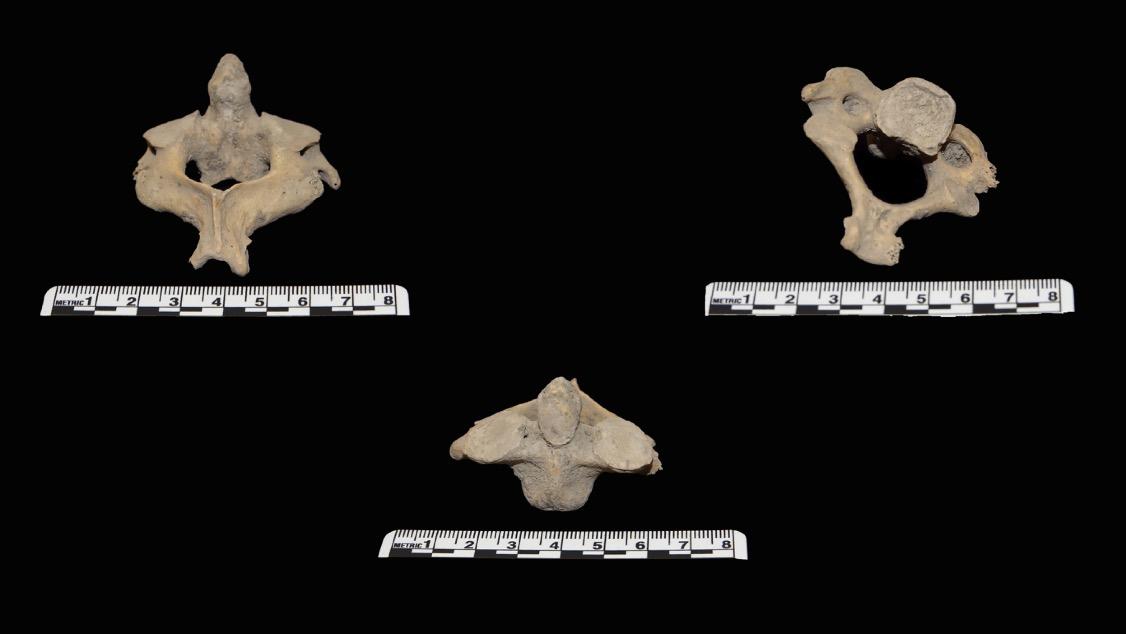


**Fig S1.** TPIT1-523 in posterior (top left), anterior (bottom middle), and inferior (top right) views.

### *TPIT1-398:* A complete, typical (C3-C6) adult cervical vertebra (Fig. S2). The bone is well preserved and only exhibits minimal postmortem trabecular exposure. Degenerative joint disease (DJD) is mostly present on the left side of the element: the surface of the left superior articular facet exhibits porosity and osteophytic growth, which may be indicative of the degradation of the cartilage disc with the superior vertebra. Possibly a compensatory effect, the left portion of the bifurcated spinous process shows more robusticity than the right, and the uncinate process of the cervical body extends more on the left than on the right. The length and width of the left superior articular facet are 20.02 mm and 15.27 mm, respectively. In contrast, the length and width of the right superior articular facet are 19.14 mm and 12 mm, respectively.


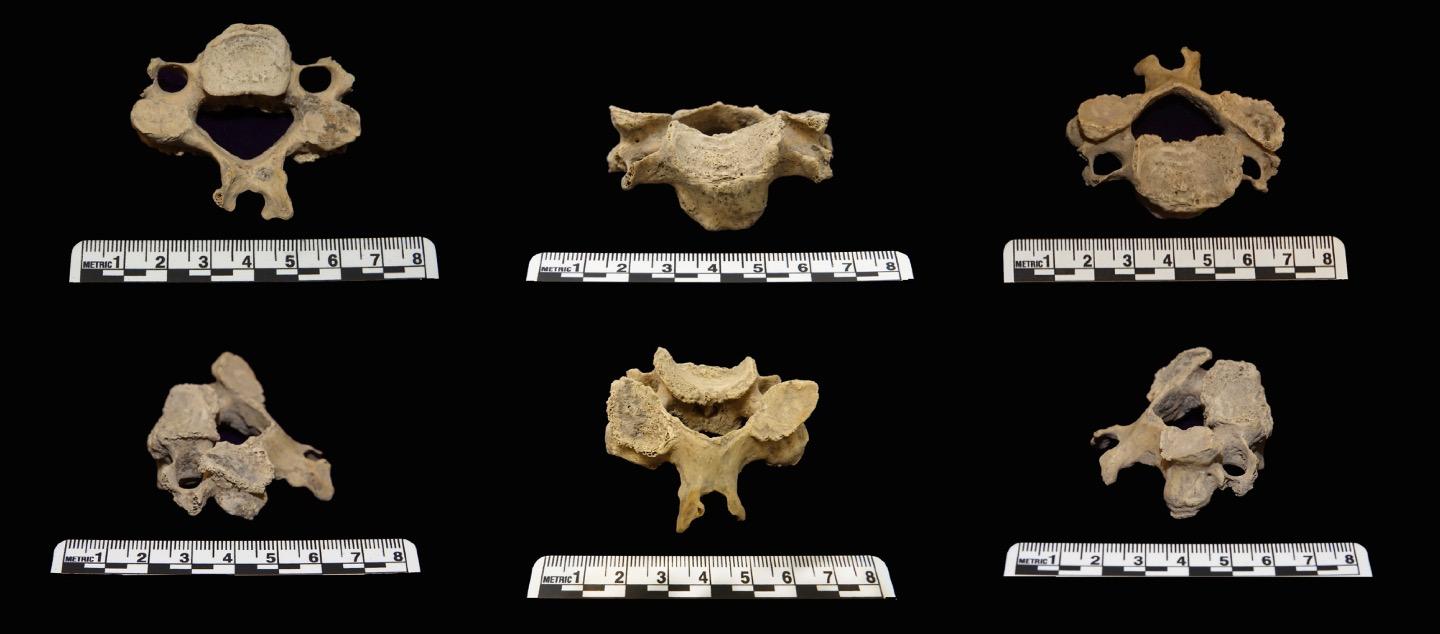


**Fig S2.** TPIT1-398 in inferior (top left), anterior (top middle), superior (top right), left lateral (bottom left), posterior (bottom middle), and right lateral (bottom right) views.

### *TPIT1-42:* A subadult, typical (T2-T9) thoracic vertebra, most likely a lower thoracic (Fig S3). The pedicles are almost completely fused to the centrum of the vertebrae; however, evidence of fusion at the neurocentral junction is still present on the superior and inferior surfaces of the centrum. The crescentic impressions of the neurocentral junction indicate that this individual died before fusion could be fully completed. Neurocentral fusion of the thoracic region tends to be the final one to be completed, the lumbar region being the first one to begin neurocentral fusion [(Baker et al., 2005)](https://www.zotero.org/google-docs/?XpvJKr). The fusion of the neurocentral junction of the thoracic vertebrae begins around the age of two, and fusion will be finalized between the ages of five and six [(Scheuer and Black, 2000)](https://www.zotero.org/google-docs/?Oy069n). Based on the features of this specimen, this bone belonged to an individual who was younger than six years of age. The right transverse process, the spinous process, and the anterior portion of the centrum are missing postmortem.


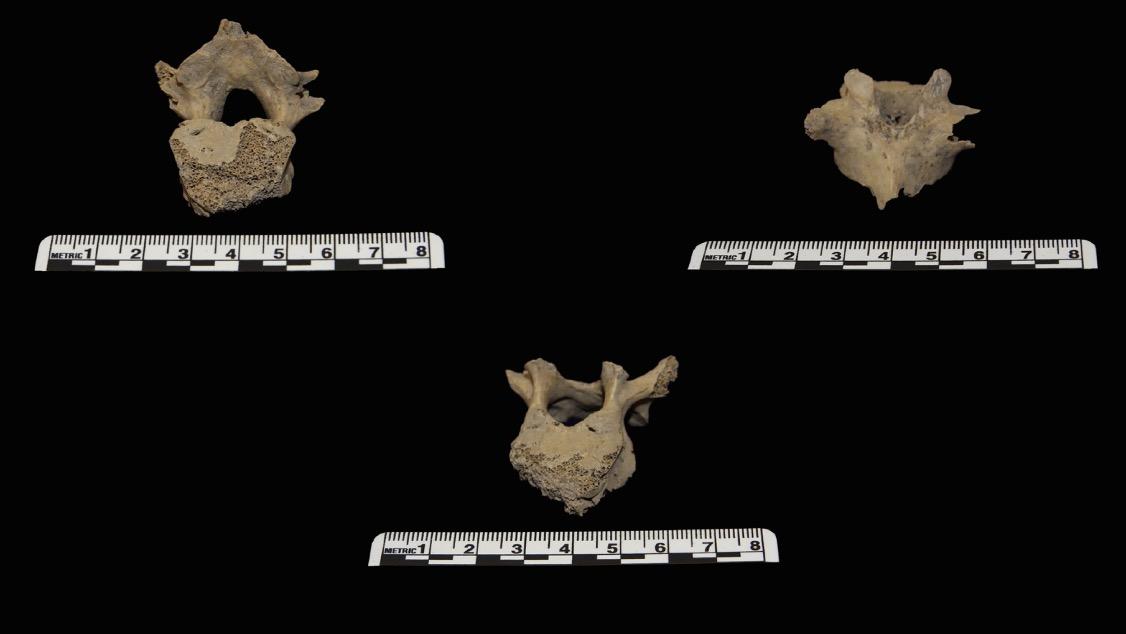


**Fig. S3.** TPIT1-42 in inferior (top left), anterior (bottom middle), and posterior (top right) views.

### *TPIT1-39***:** A mostly complete, lower lumbar vertebra of a nonadult (Fig. S4). Based on the rectangular shape of the lamina of this specimen, it is highly probable that it is the ultimate lumbar vertebrae (L5). Postmortem damage is evident by the exposure of the trabecular bone around the anterior rim of the superior aspect of the centrum, as well as breakage of the transverse and spinous processes. The superior and inferior surfaces of the centrum exhibit signs of billowing, a diagnostic feature of nonadults. The neurocentral junction appears completely fused, which usually begins in the lumbar region between the second year of life and is completed by the fourth or fifth year (Scheuer and Black, 2000). Based on this, this individual was at least five years of age.


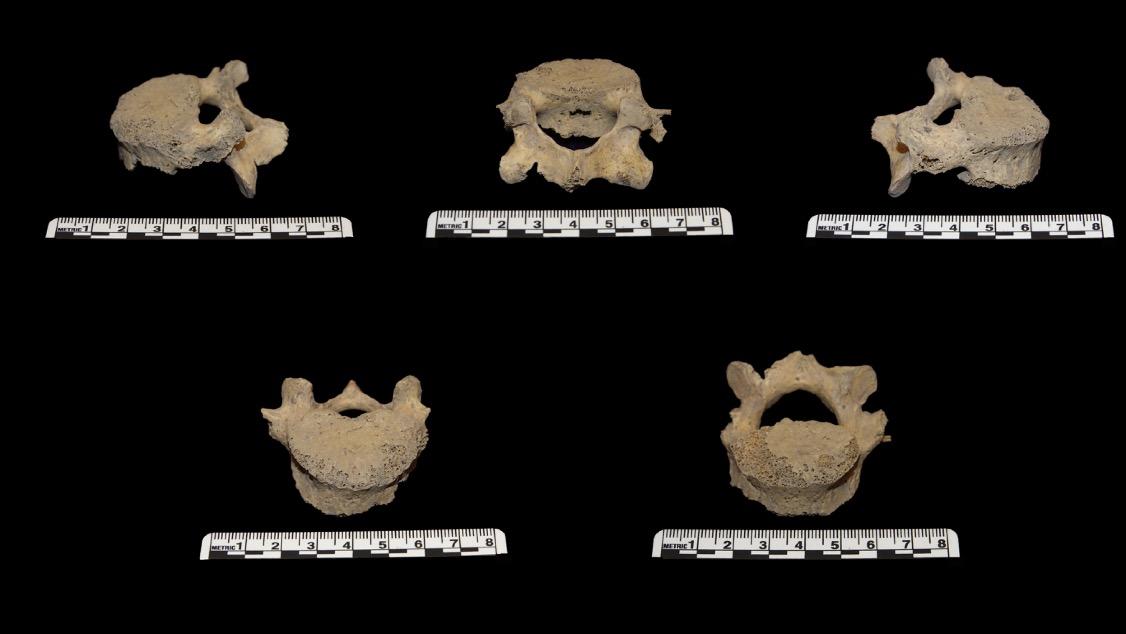


**Fig S4.** TPIT1-39 in left lateral (top left), posterior (top middle), right lateral (top right), superior-anterior (bottom left), and inferior-anterior (bottom right) views.

### *TPIT1-178:* A partial lumbar vertebra of an adult (Fig. S5). While most of the anterior portion of the vertebra is missing postmortem, the remaining bone exhibits bilateral spondylolysis. Spondylolysis tends to primarily occur in the ultimate lumbar vertebrae (L5) but can also occasionally occur in other lumbar segments, and this condition has been linked to the hyperextension of the lower back [(Ward and Latimer, 2005)](https://www.zotero.org/google-docs/?Cn3ZDT). This is the only vertebra that presents evidence of this condition, and due to the absence of its vertebral body, it cannot be inferred if this individual also suffered from spondylolisthesis or other spinal deformities. This specimen presents evidence of healing at the *pars interarticularis*.


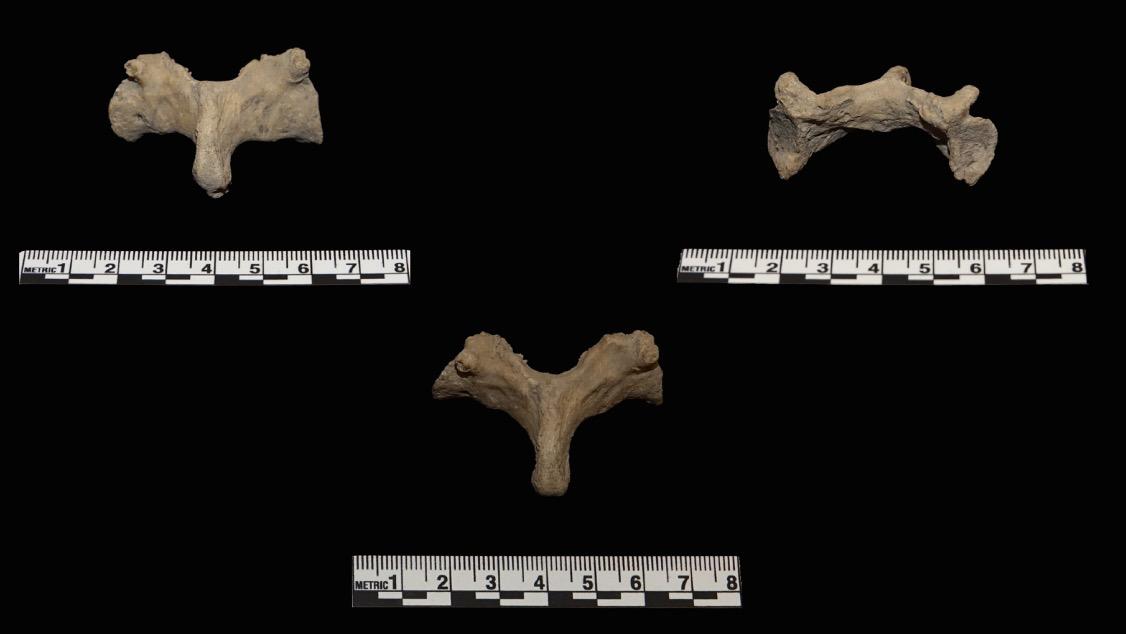


**Fig. S5.** TPIT1-178 in anterior (top left), supero-anterior (middle), and posterior (top right) views.

### *TPIT1-NC:* An adult, lower thoracic vertebra (Fig. S6). The transverse and spinous processes are broken postmortem. Exfoliated cortical bone of the superior vertebral body has eroded due to taphonomy. Based on the complete costal facets, this is a lower thoracic vertebra (T10-12). The costal facets are low on the vertebral body, which suggests that this specimen could be T11 or T12. Typically, the inferior articular facets of T12 assume the lumbar pattern and are convex and sagittally oriented, whereas the superior articular facets are planar and have a coronal orientation [(Forseen et al., 2015)](https://www.zotero.org/google-docs/?3SJLXW). The superior and inferior articular facets of this specimen follow the typical thoracic pattern, which indicates that this vertebra is T11.


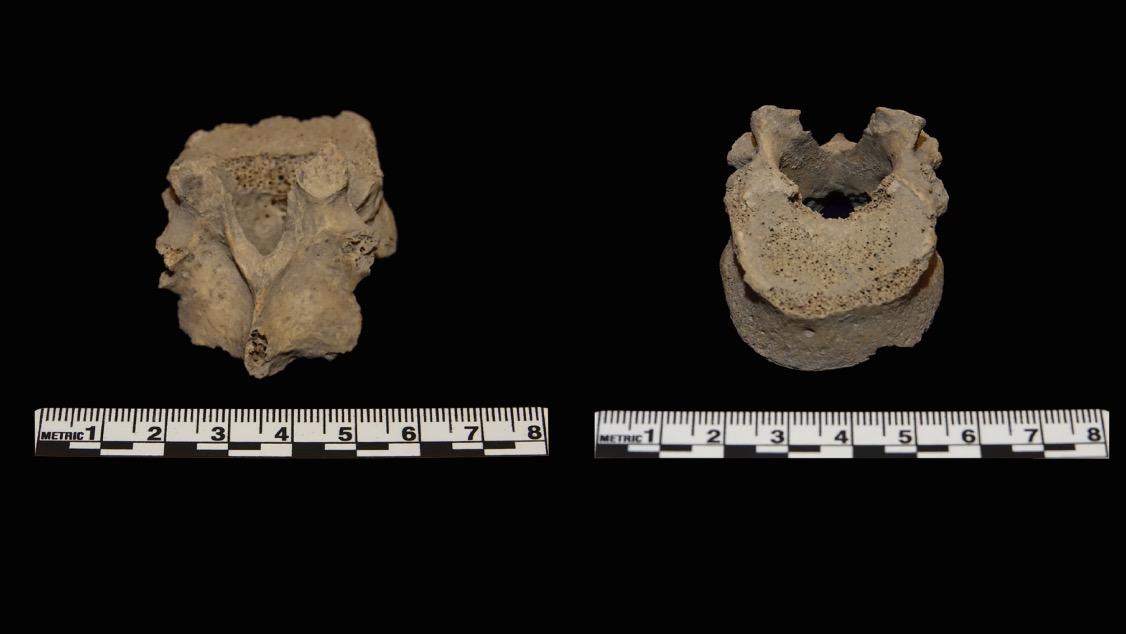


**Fig S6.** TPIT1-NC in supero-posterior (left) and supero-anterior (right) views.

### *TPIT1-473*: A complete right first rib (R1) of a nonadult (Fig S7). The rib head shows evidence of incomplete fusion. The epiphyses for the heads of ribs are usually the last of the rib epiphyses to form, and they complete fusion by the age of 17. Fusion typically begins in the upper and lower ribs first, completing by 22-25 years (Scheuer and Black, 2000). Based on the characteristics of TPIT1-473, the individual was younger than 17 years of age.


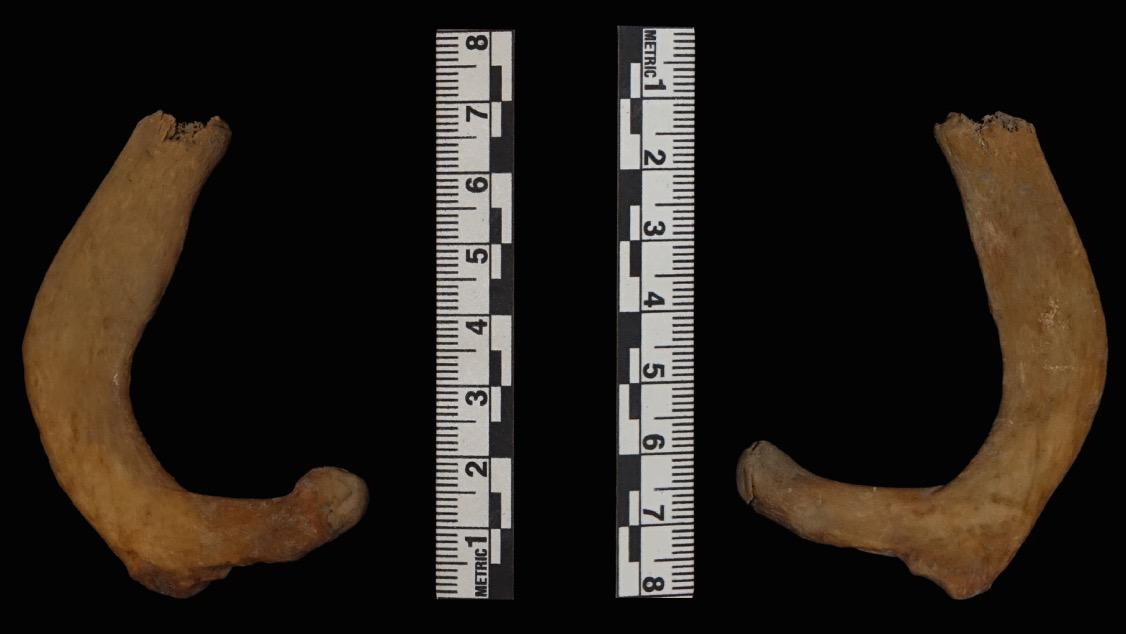


**Fig S7.** TPIT1-473 in superior (left) and inferior (right) views.

### *TPIT1-36:* A complete, adult right rib 12 (Fig. S8). This bone is complete, and the maximum length is 9.1 cm.


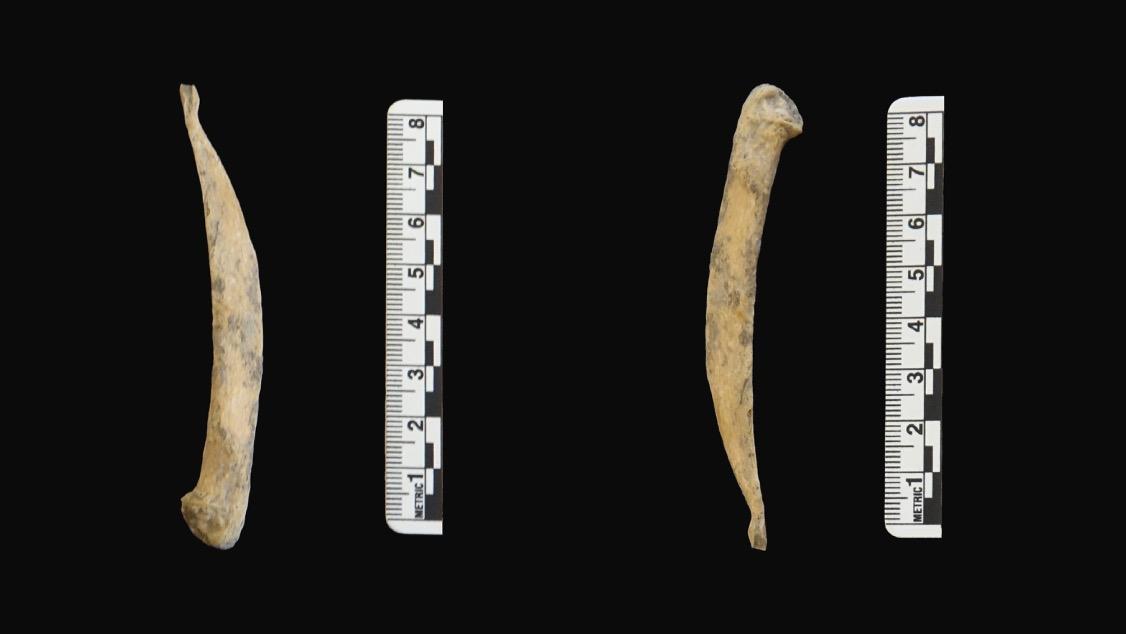


**Fig S8.** TPIT1-36 in anterior (left) and posterior (right) views.

###

### *TPIT1-14:* A right, typical rib of an adult (Fig. S9). The rib head and the sternal end of this specimen have broken postmortem.


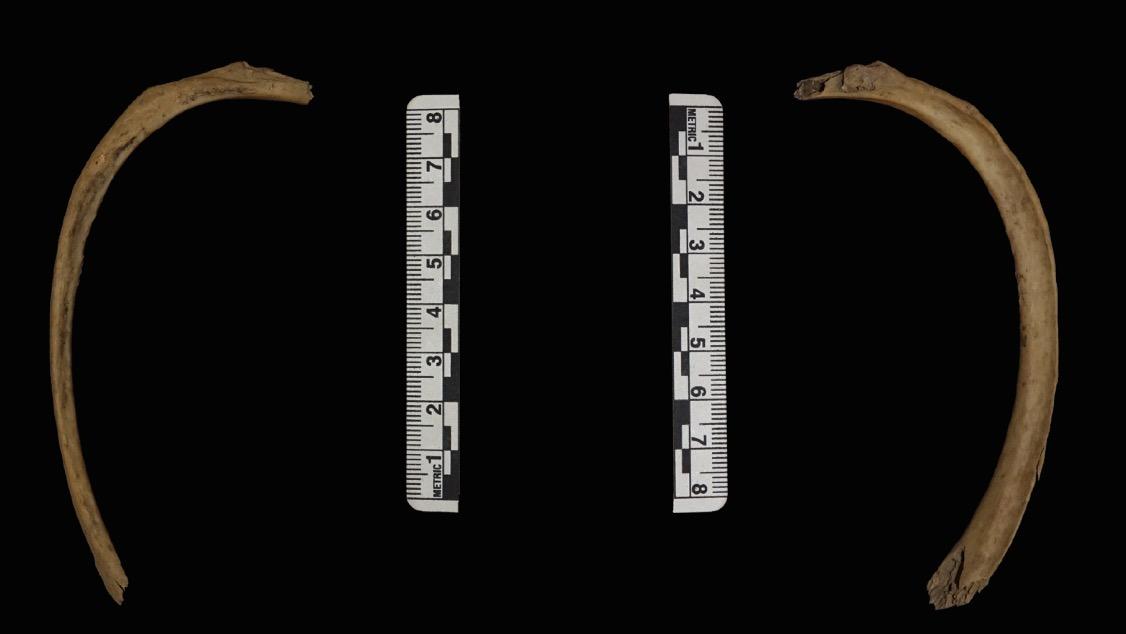


**Fig S9.** TPIT1-14 in superior (left) and inferior (right) views.

### *TPIT1-49:* A fragmented right typical rib (Fig. S10). Only the costal angle is preserved. A small bony callus on the caudal border may be evidence of a healed antemortem fracture.


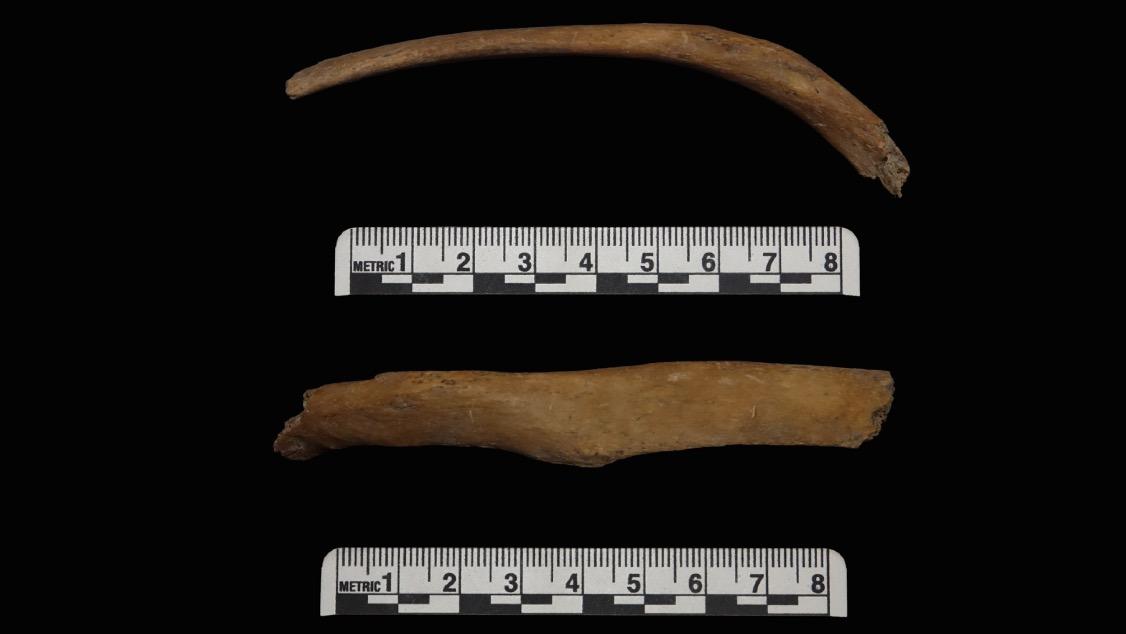


**Fig S10.** TPIT1-49 in superior (top) and lateral (bottom) views.

### *TPIT1-318:* A fragmented left typical rib (Fig. S11). The rib head, tubercle, and the sternal end are missing postmortem.


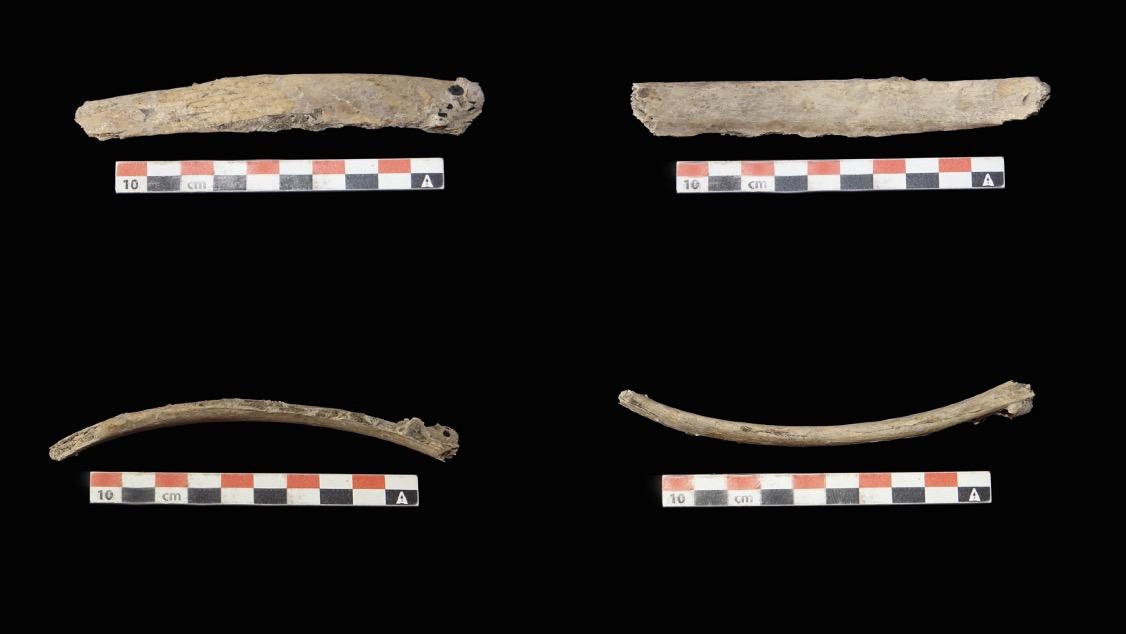


**Fig S11.** TPIT1-318 in anterior (top left), posterior (top right), inferior (bottom left), and superior (bottom right) views.

### *TPIT1-326:* A left typical rib of a nonadult (Fig. S12). The bone is well preserved, showing the billowed sternal end of a juvenile, and incomplete fusion of the tubercles and vertebral epiphysis.

###


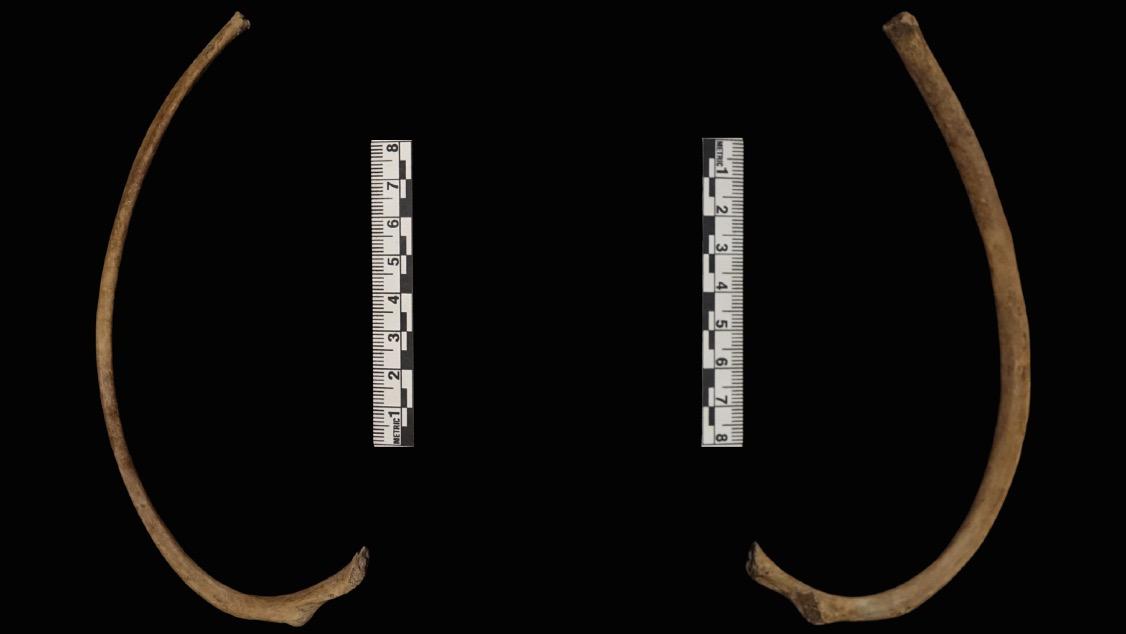


**Fig S12**. TPIT1-326 in superior (left) and inferior (right) views.

### *TPIT1-332:* A left typical rib of an adult (Fig. S13). The rib is broken at the midshaft. The break matches on both pieces and the bones can be refitted. The sternal end of the rib broke postmortem.


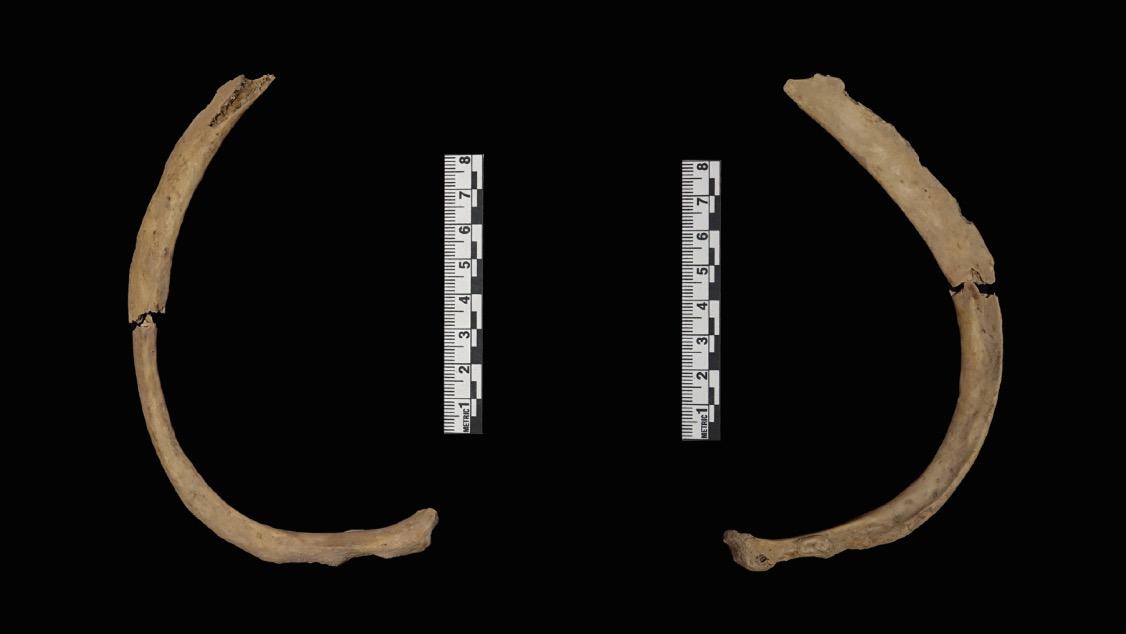


**Fig S13.** TPIT1-332 in superior (left) and inferior (right) views.

### *TPIT1-336*: A fragmented left, typical rib of an adult (Fig. S14). Only the rib head and tubercle are preserved.


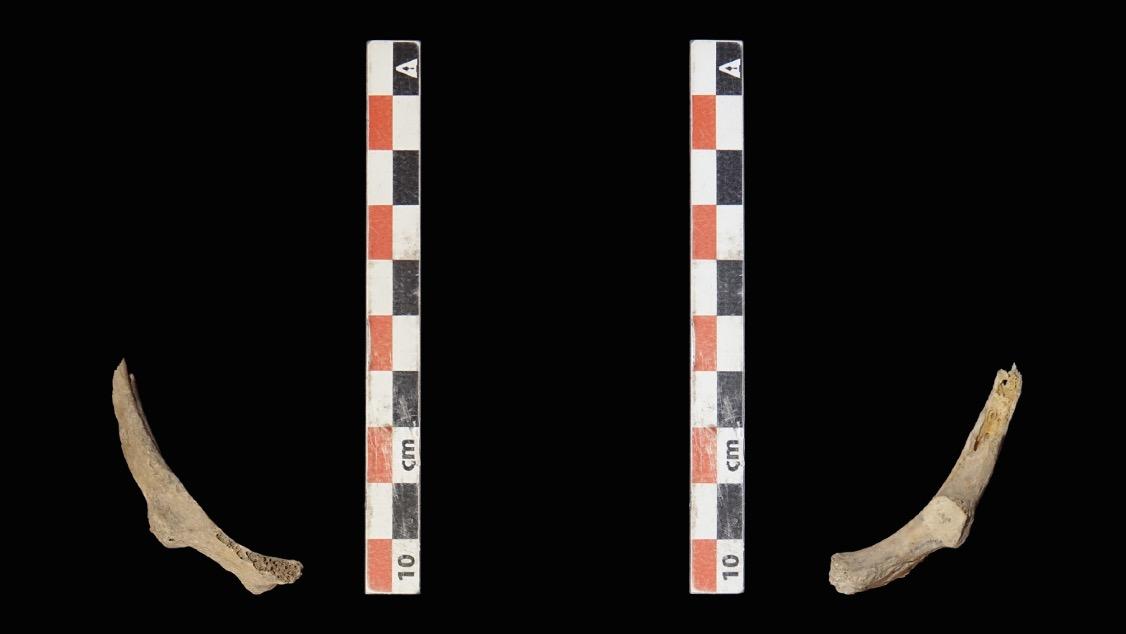


**Fig S14**. TPIT1-336 in superior (left) and inferior (right) views.

### *TPIT1-376:* A fragmented left typical rib shaft of an adult (Fig. S15). The rib head, tubercle, and the distal portion of the rib shaft are missing postmortem.


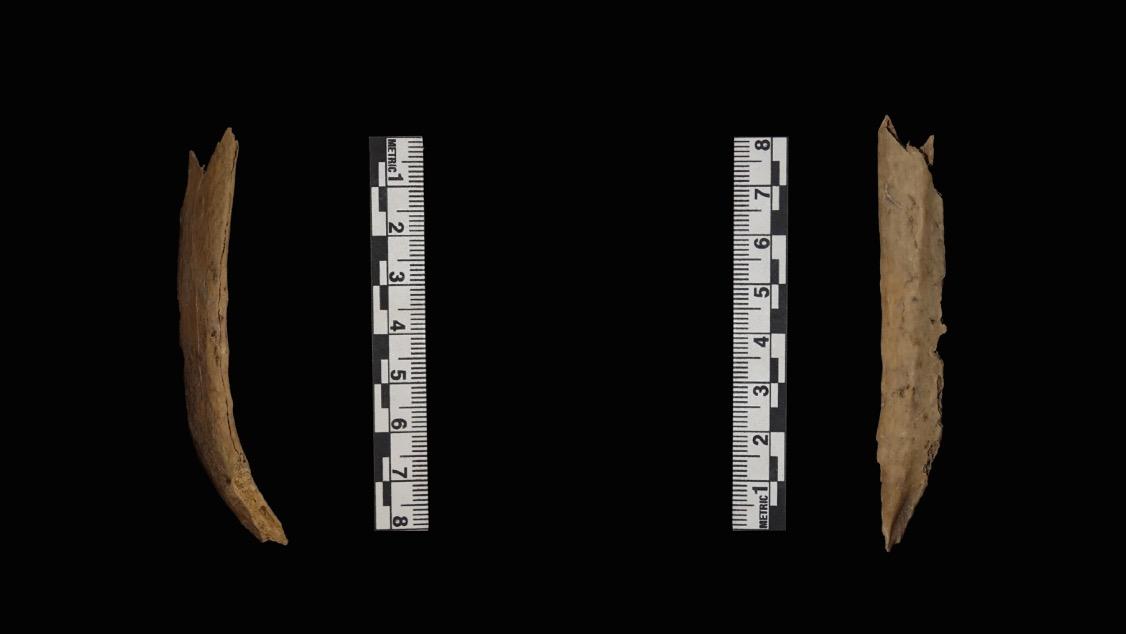


**Fig S15.** TPIT1-376 in superior (left) and internal (right) views.

### *TPIT1-426:* A right typical rib of an adult (Fig. S16). The sternal half of the rib shaft is missing.


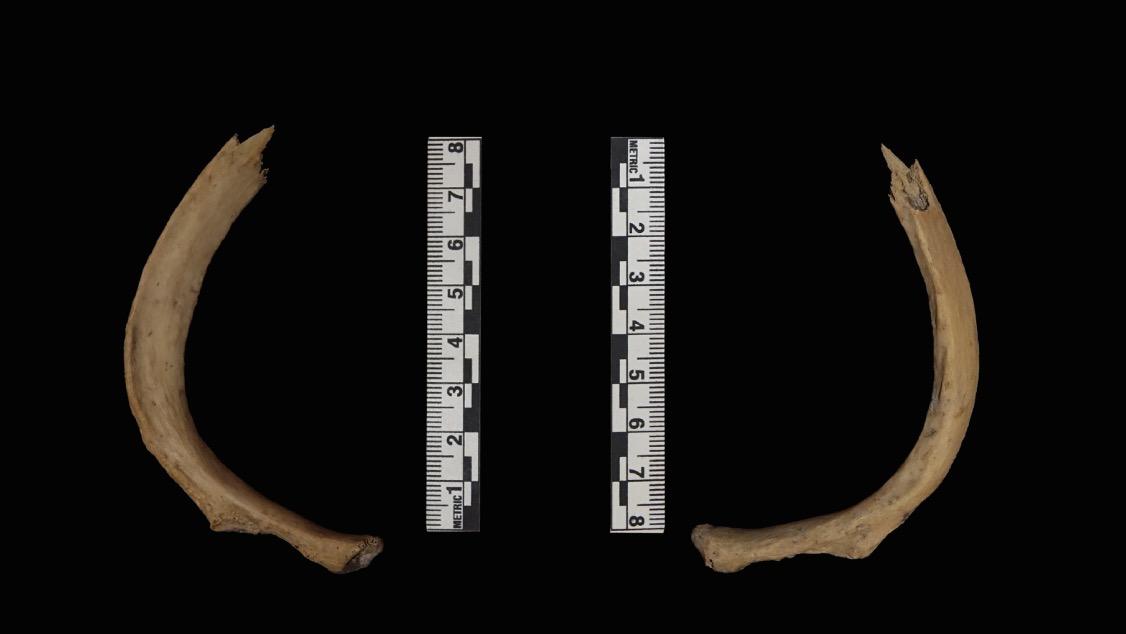


**Fig S16.** TPIT1-426 in inferior (left) and superior (right) views.

###

### *TPIT1-446:* A right typical rib of an adult (Fig S17). The rib head and the anterior portion of the shaft are missing from this specimen.


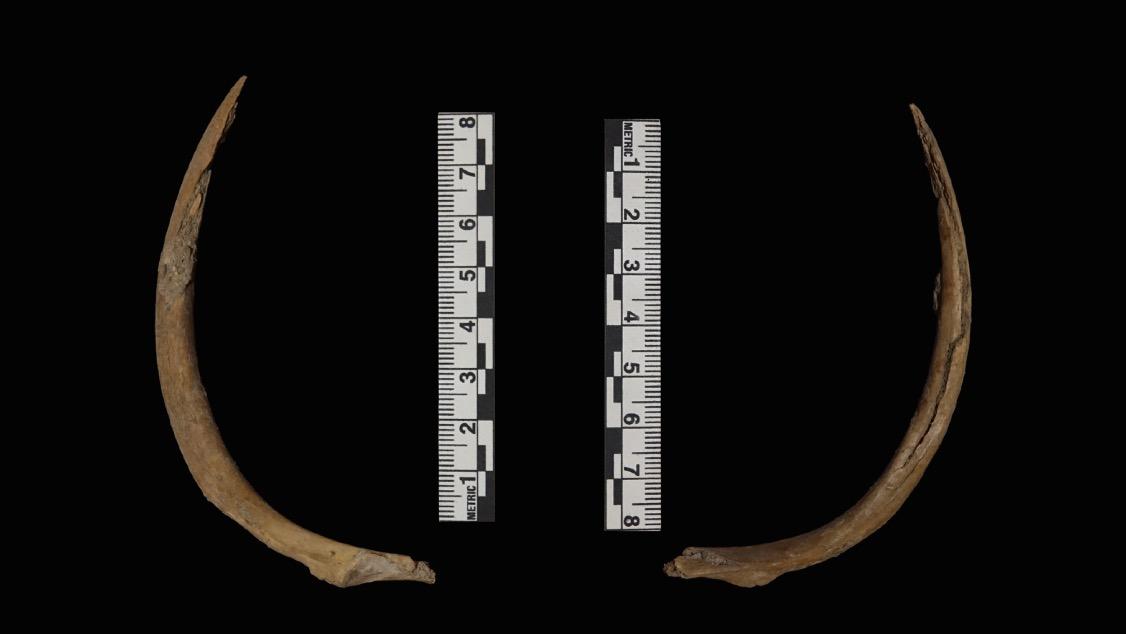


**Fig S17.** TPIT1-446 in inferior (left) and superior (right) views.

###

### *TPIT1-389:* A fragmented right rib of an adult (Fig. S18). The head and costal angle of the rib are present but obscured by a significant healed bony callus. The appearance is consistent with a healed fracture, which likely was not properly set after sustained trauma. The location of the lesion at the vertebral end suggests that the individual sustained this injury on their back.

###


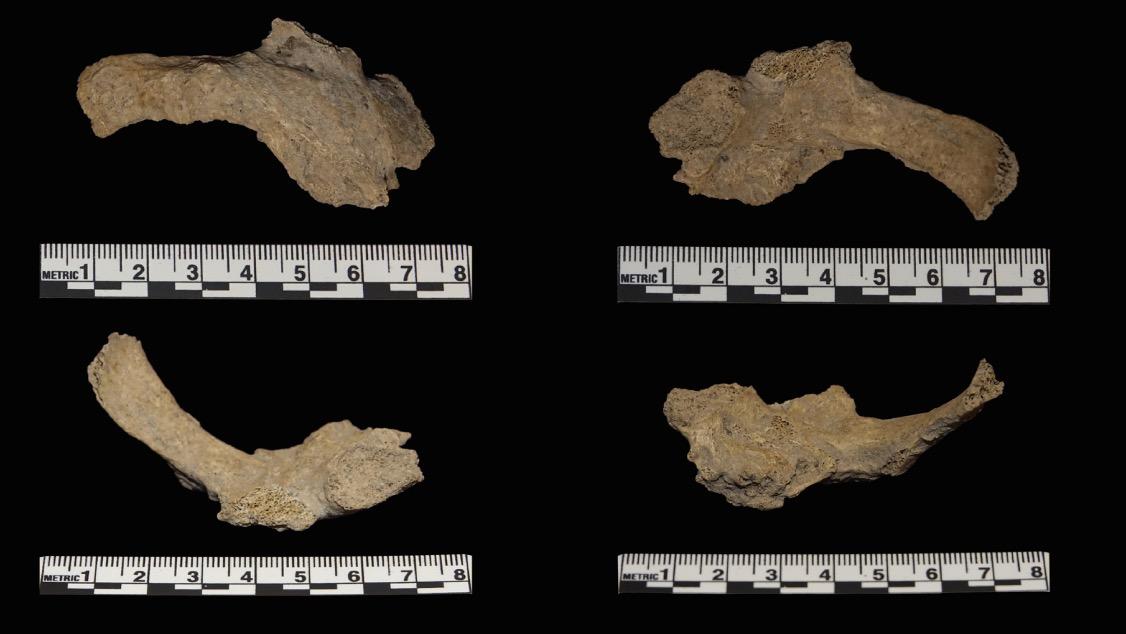


**Fig S18**. TPIT1-389 in anterior (top left), posterior (top right), superior (bottom left), and inferior (bottom right) views.

###

### *TPIT1-532:* A right typical rib of an adult (Fig S19). The sternal end is missing postmortem.


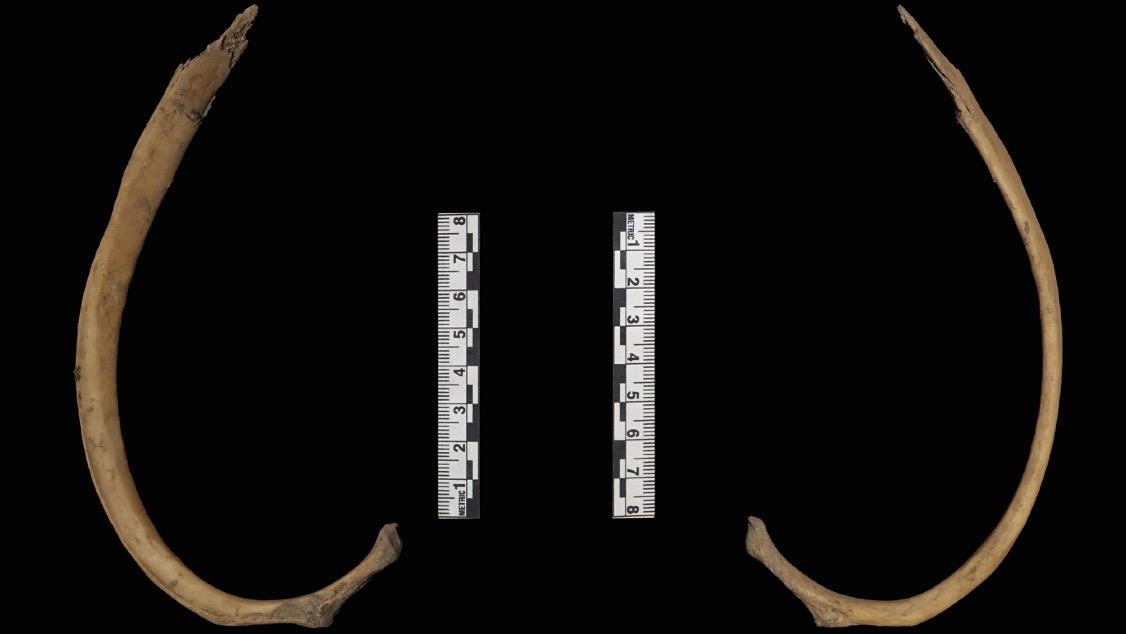


**Fig S19**. TPIT1-532 in inferior (left) and superior (right) views.

##

## **CLAVICLES & SCAPULAE**

### *TPIT1-44:* A right clavicle (Fig. S20) of a young adult, based on the unfused sternal end, which initiates fusion in the late 20s (Scheuer and Black, 2000). This exact feature on this surface is difficult to fully assess due to the presence of sediment on the sternal end. The maximum length is 14.4 cm, the anteroposterior diameter at midshaft is 11.24 mm, and the superoinferior diameter at midshaft is 9.34 mm.


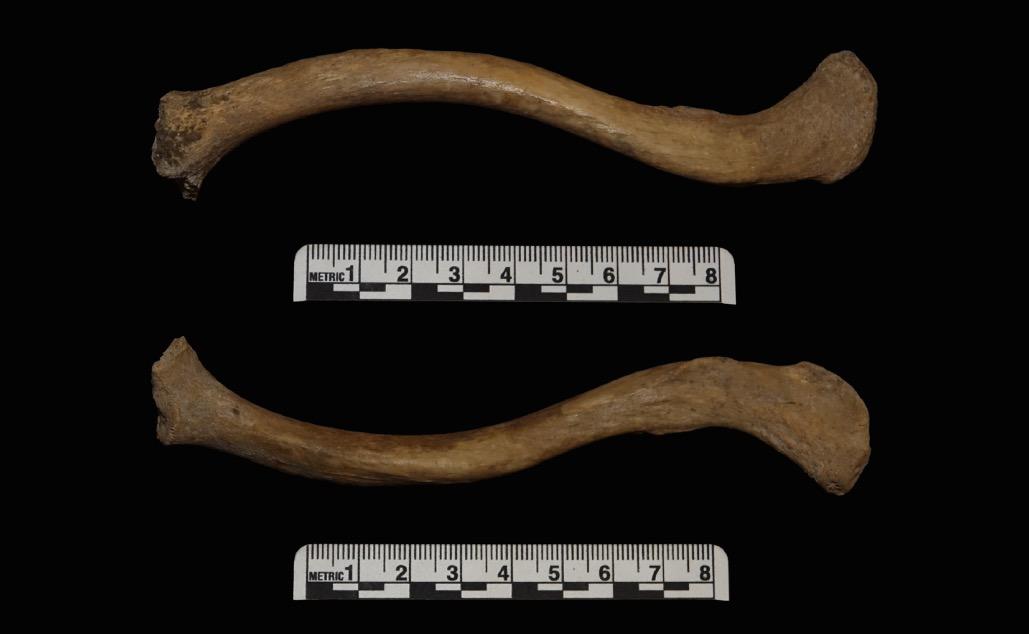


**Fig S20.** TPIT1-44 in superior (top) and inferior (bottom) views.

### *TPIT1- 319:* A right clavicle (Fig. S21), with the sternal end missing postmortem. There is a small focalized area of carnivore gnawing on the inferior surface near the midshaft and on the superior surface near the sternal end.


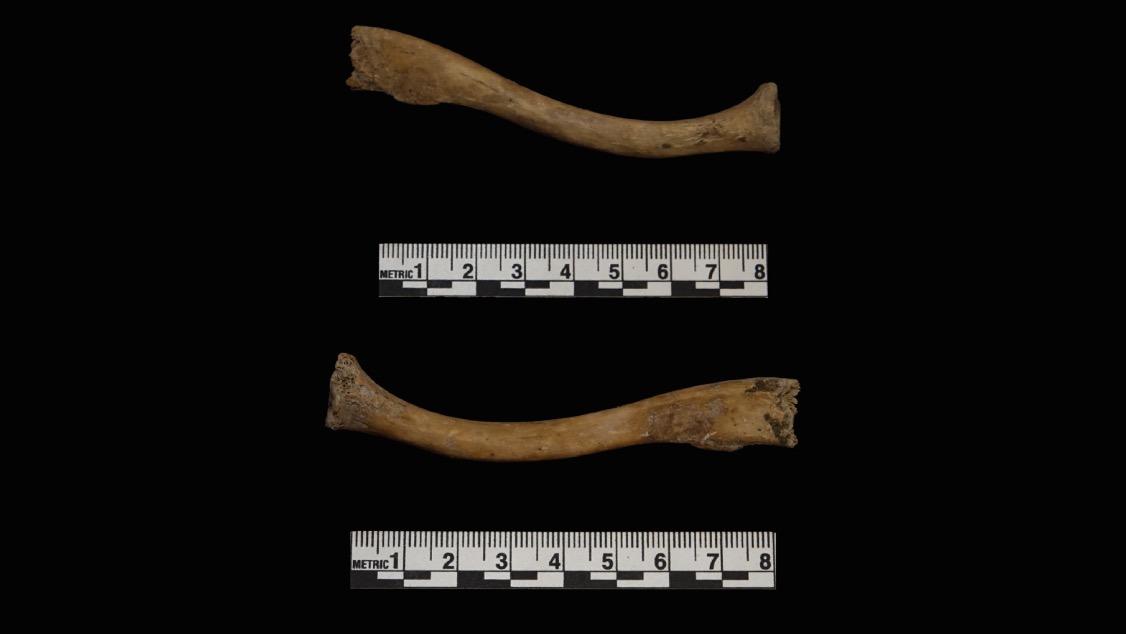


**Fig S21.** TPIT1-319 in inferior (top) and superior (bottom) views.

###

### *TPIT1-330:* A left clavicle (Fig S22) broken at the acromial and sternal ends. At midshaft, the anteroposterior diameter is 13.22 mm and the superoinferior diameter is 8.28 mm.


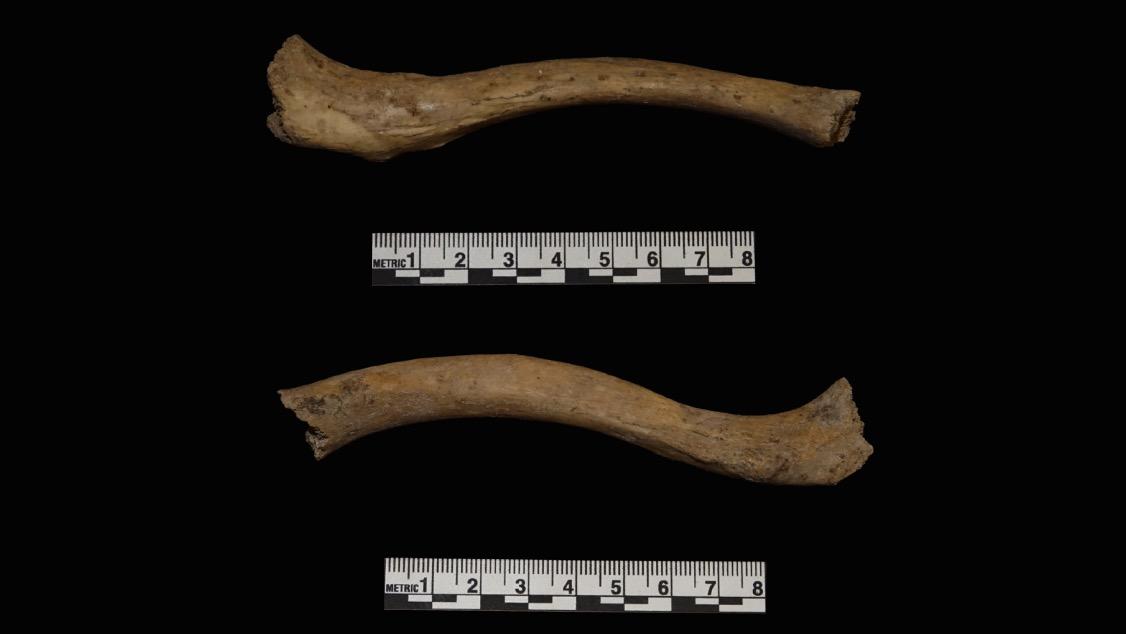


**Fig S22**. TPIT1-330 in superior (top) and inferior (bottom) views.

### *TPIT1-37:* A right, scapula fragment (Fig S23). Based on the preserved morphology, this is a fragment of the lateral border of a scapula.


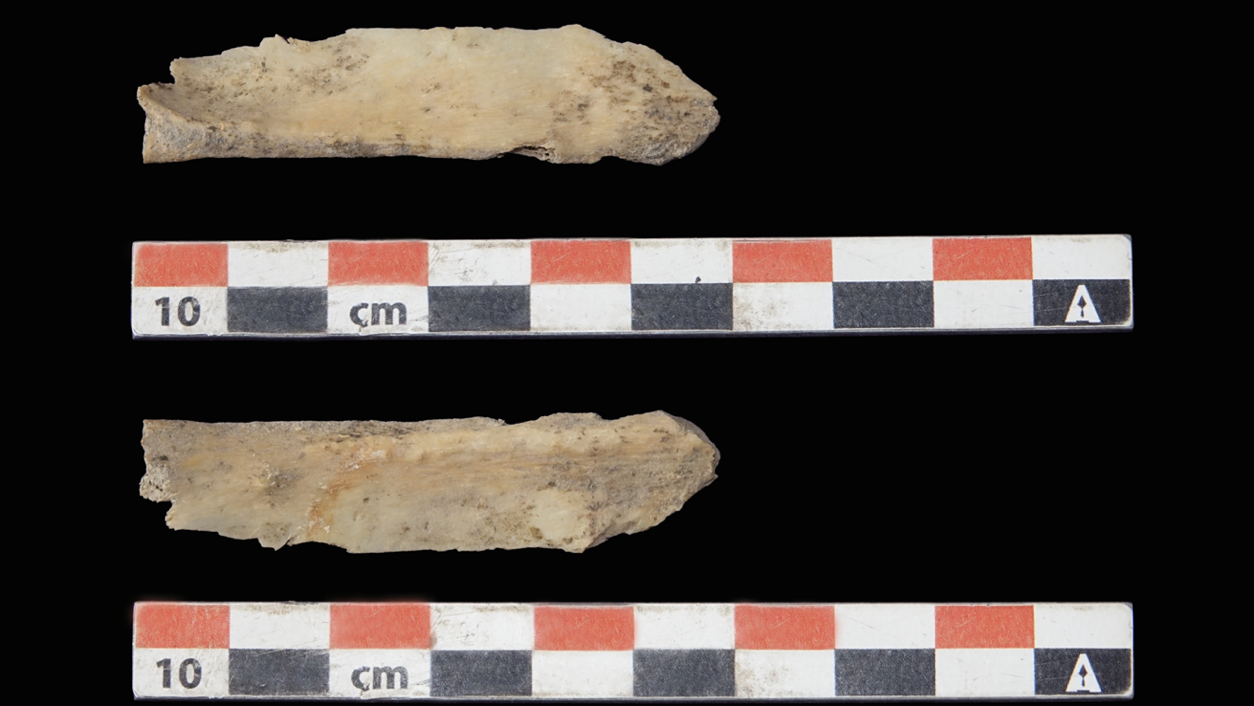


**Fig S23.** TPIT1-37 in anterior (top) and posterior (bottom) views.

### *TPIT1-405:* A left scapula (Fig S24) at the inferior angle. Part of the lateral border and muscle attachment of the teres major are preserved.

.
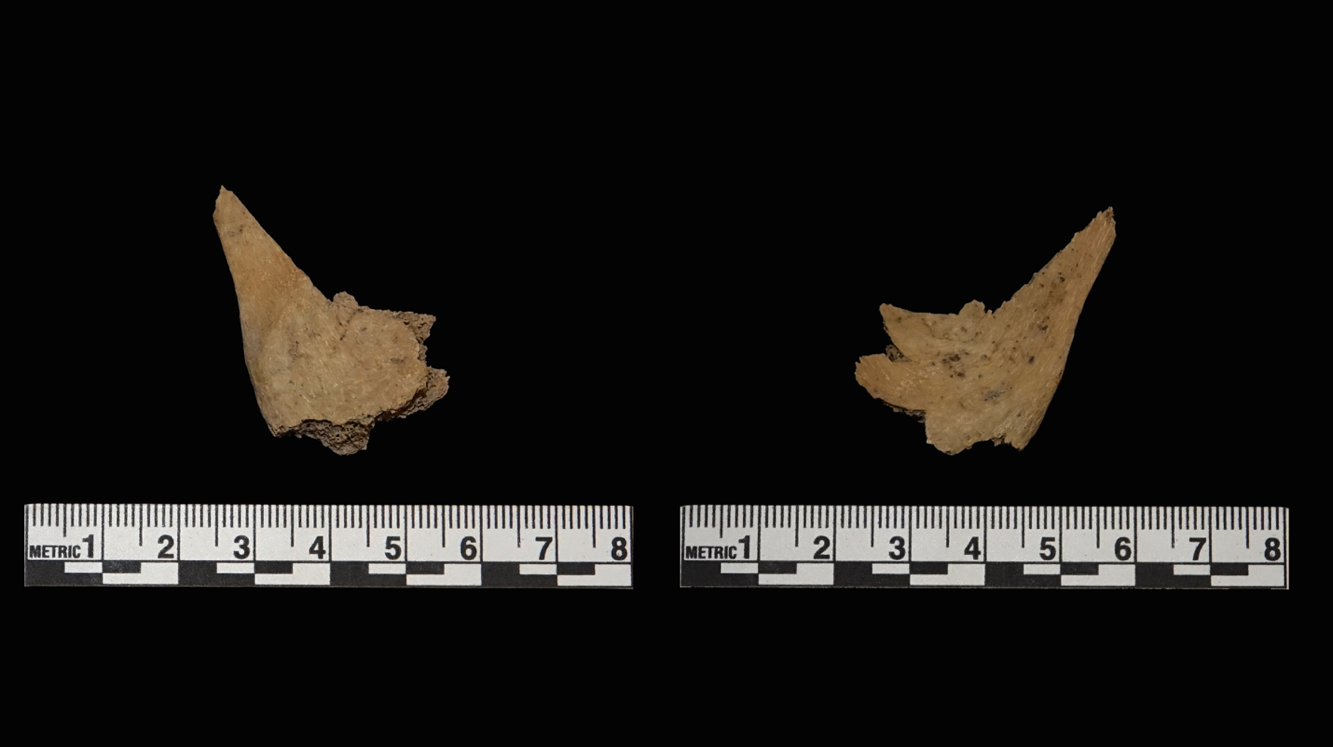


**Fig S24.** TPIT1-405 in posterior (left) and anterior (right) views.

### *TPIT1-341:* A fragmented scapula (Fig S25). Based on the landmarks, this is a fragment of the superomedial portion of the scapular body. The extent of fragmentation of this specimen makes it difficult to side with this element.


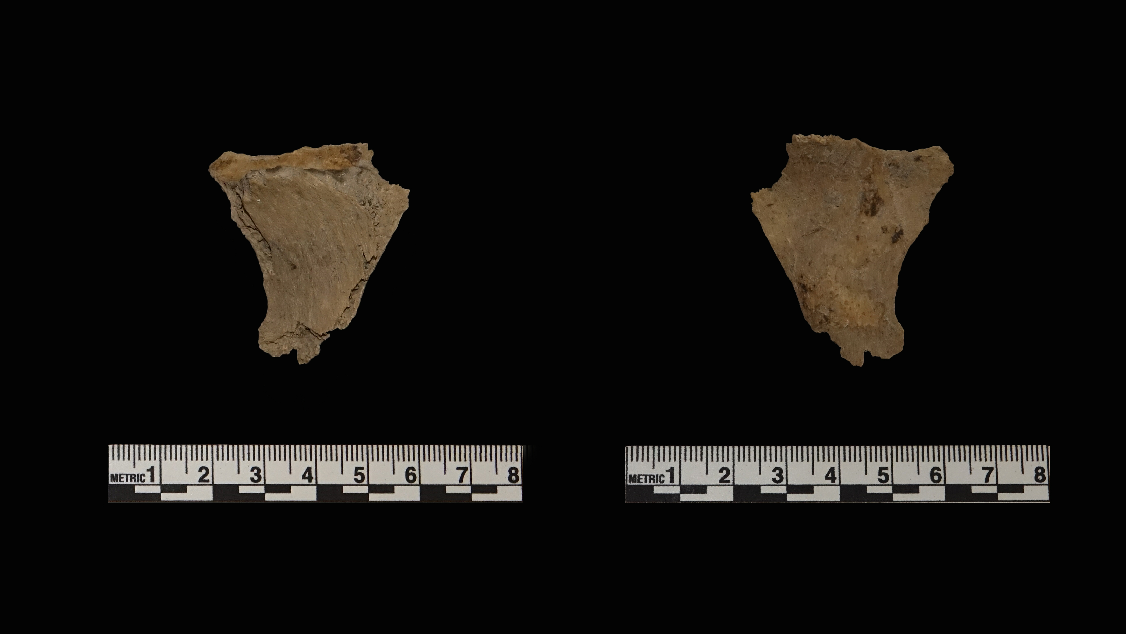


**Fig S25.** TPIT1-341 in posterior (left) and anterior (right) views.

## **LONG BONES**

### *TPIT1-40:* A complete, adult right humerus (Fig S26). The bone is well preserved, with no obvious postmortem damage. While the bone shaft is gracile, it exhibits robust entheseal changes at the deltoid tuberosity. The following measurements were taken: Maximum length = 239 mm; epicondylar length: 2.36 mm; vertical head diameter = 41.71 mm; maximum diameter at midshaft = 20.35 mm; minimum diameter at midshaft = 16.89 mm.


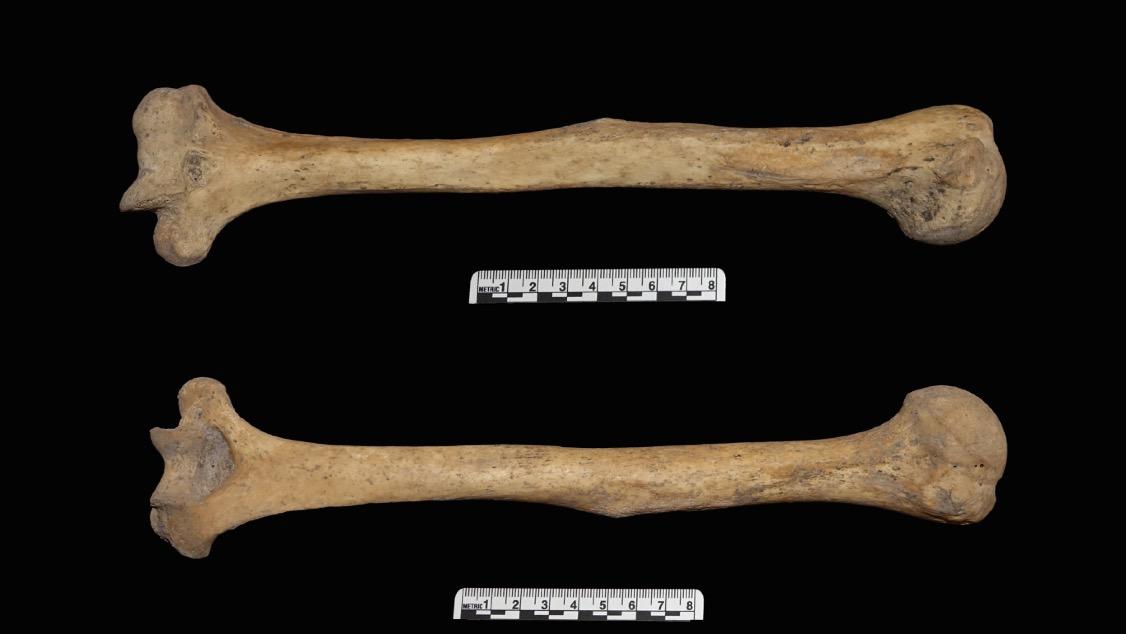


**Fig S26.** TPIT1-40 in anterior (top) and posterior (bottom) views.

## **OS COXAE**

### *TPIT1-491:* A mostly complete, right ilium of a nonadult (Fig S27). The majority of the iliac crest epiphyseal surface is missing postmortem. The associated ischium and pubis were not recovered; the acetabular epiphysis between the pubis and the ilium begins fusing around the ages of nine and ten. The maximum iliac length is 102 mm. Based on these data, this ilium likely belongs to a child of 6-9 years.

###
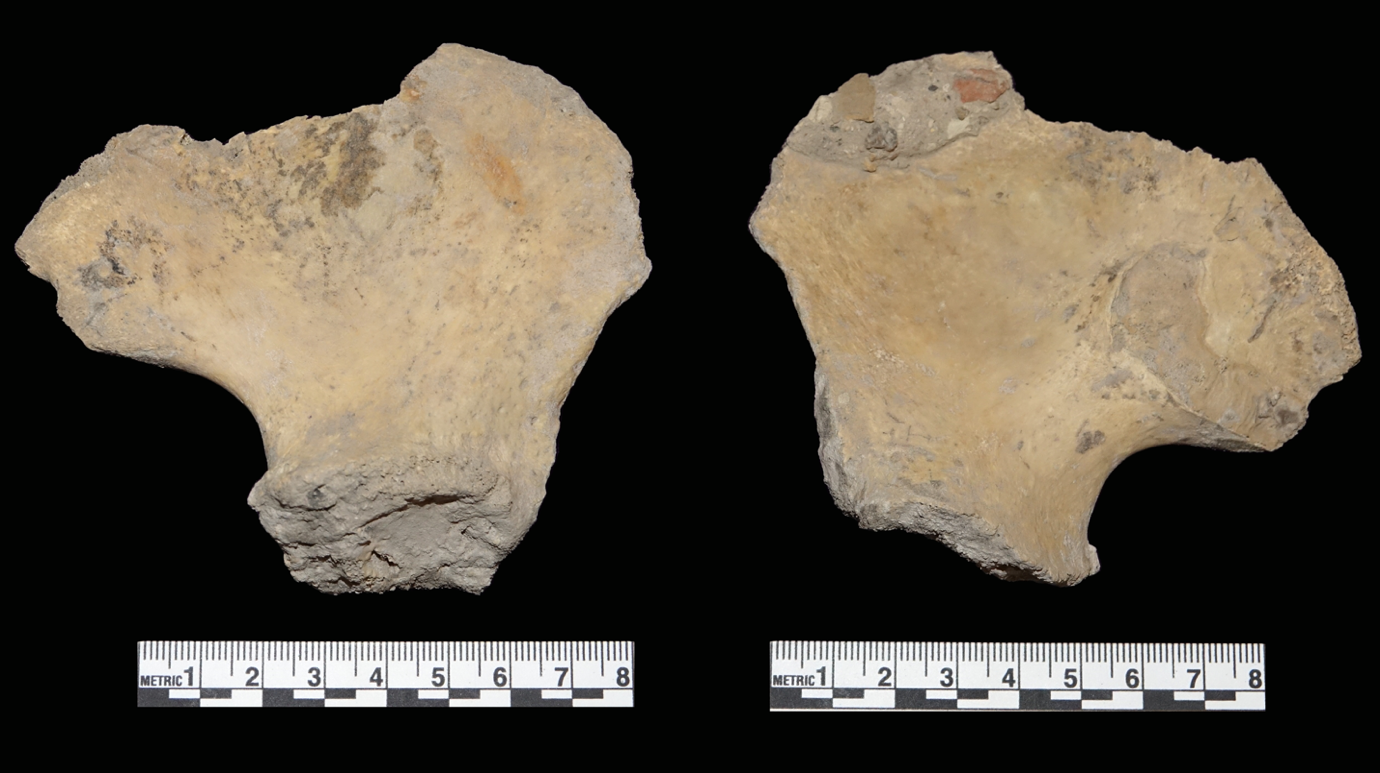


**Fig S27.** TPIT1-491 in lateral (left) and medial (right) views.

### *TPIT1-51:* A mostly complete right os coxa of an adult (Fig S28). The pubic symphysis is missing postmortem, only the inferior portion, which leads into the ischiopubic ramus, remains. The inferior half of the ischial tuberosity is missing, and this half of the ischiopubic ramus is also missing. The anterior superior iliac spine is missing postmortem. Based on the narrow greater sciatic notch, broad ischiopubic ramus, and straight subpubic concavity, the probable sex of the individual was male. The obturator foramen also suggests that the individual was male; the shape of the obturator foramen is oval, which is a feature seen in males, while females tend to have a triangular obturator foramen (Fig S29) [(Ferembach et al., 1980; Rennie et al., 2023)](https://www.zotero.org/google-docs/?BGoj4l). The estimation of sex would be more confident if a ventral arc could be observed. The gluteal lines on the lateral portion of the ilium are rugose, suggesting extensive use of the gluteal muscles. Age is difficult to estimate with the sediment covering the auricular surface and the absence of the pubic symphysis.


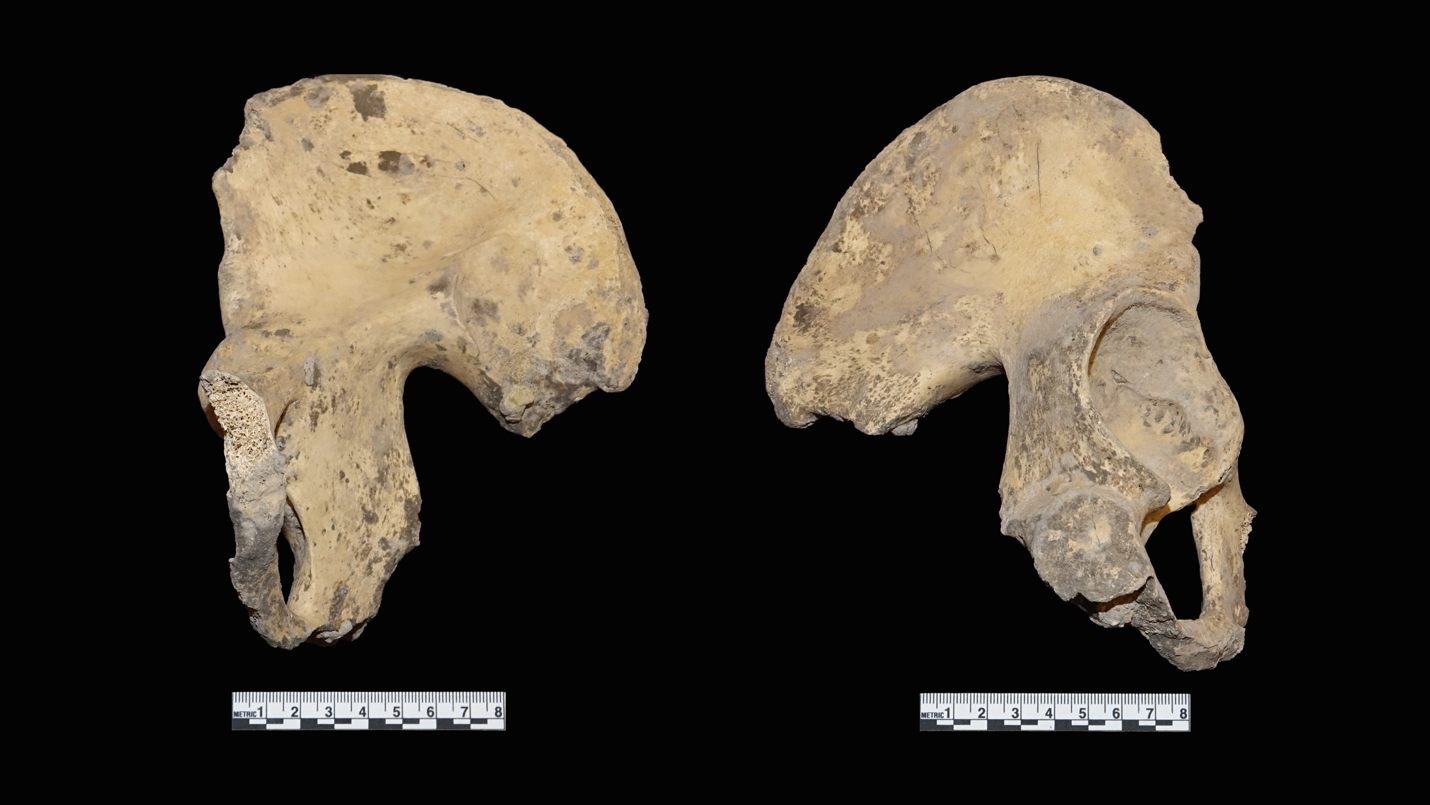


**Fig S28**. TPIT1-51 in medial (left) and latero-inferior (right) views.


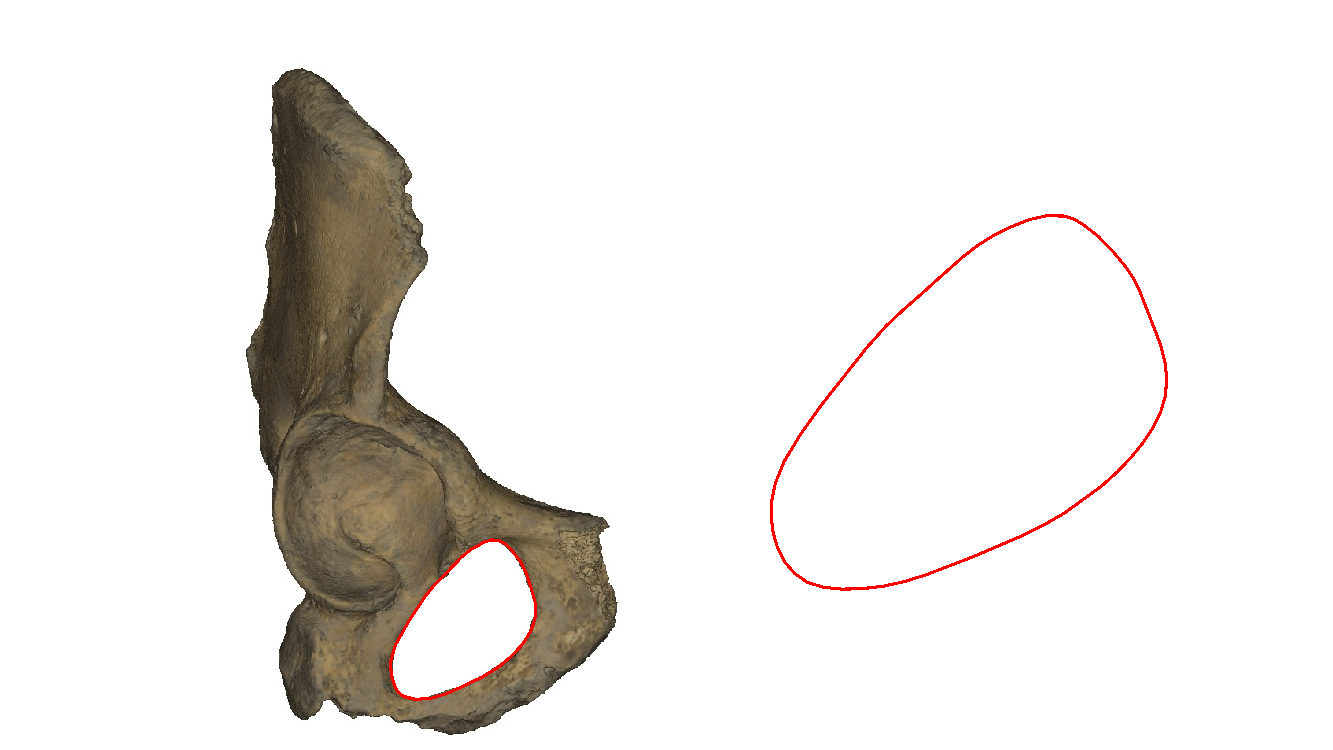


**Fig S29.** Outline of the shape of the obturator foramen of TPIT1-51.

##

## **HANDS AND FEET**

### *TPIT1-425:* A complete, adult hand proximal phalanx (Fig S30). Based on the morphology of the proximal and distal ends, this specimen is a left phalanx (ray 2-4) (Case & Heilman, 2006).


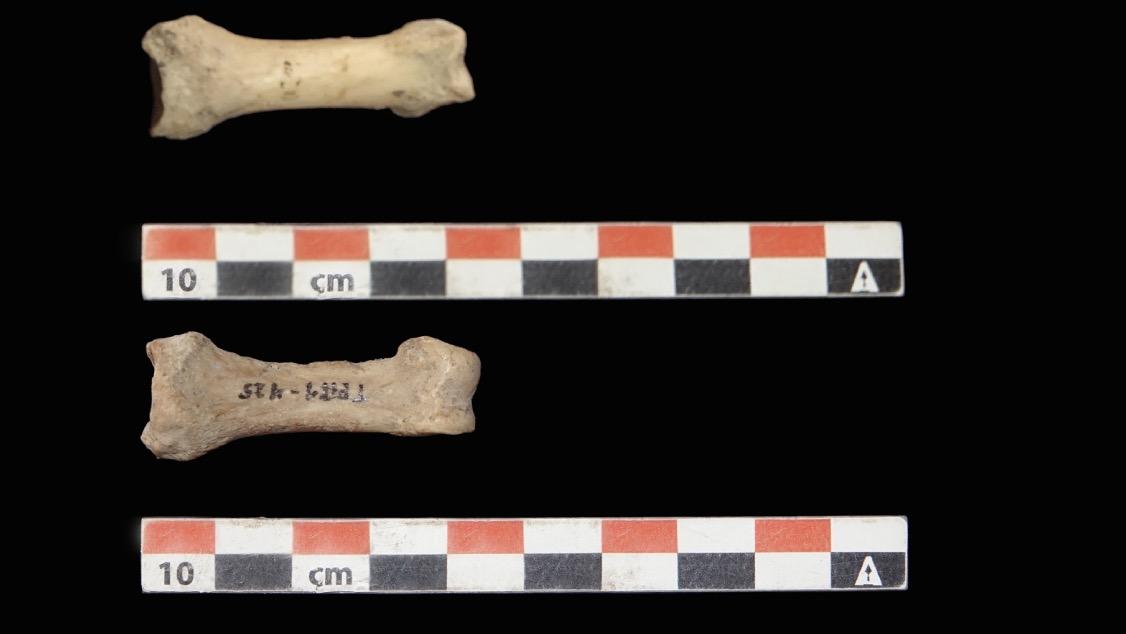


**Fig S30.** TPIT1-425 in dorsal (top) and palmar (bottom) views.

### *TPIT1-484:* A complete, adult intermediate hand phalanx (Fig S31). Based on the projection of the superior margin of the proximal facet, this is a right phalanx (ray 2-4) (Case & Heilman, 2006).


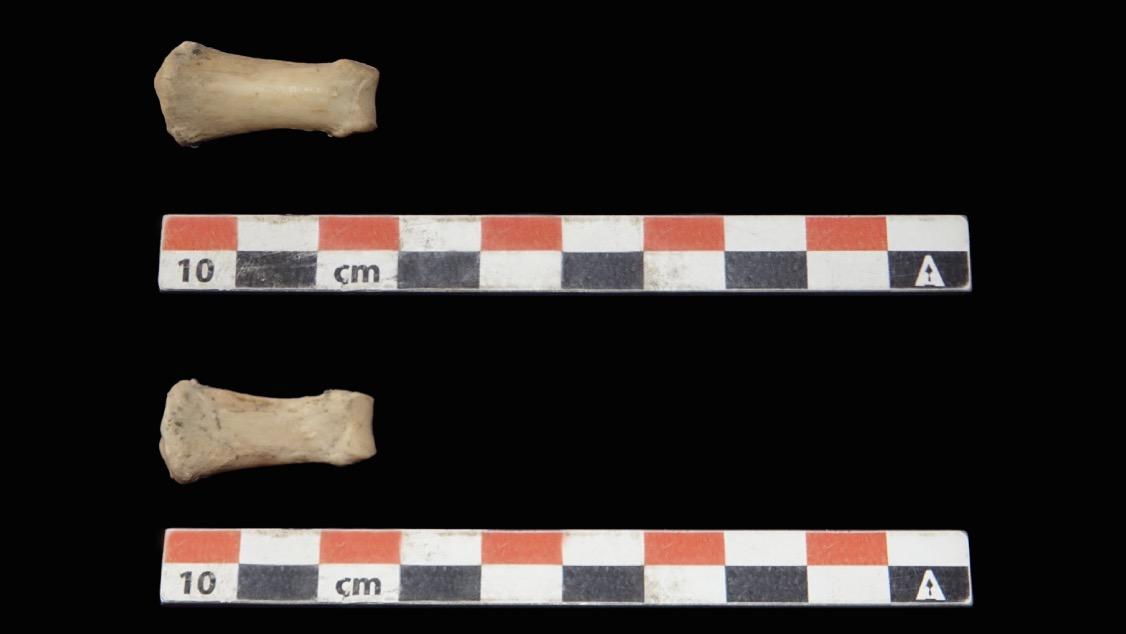


**Fig S31.** TPIT1-484 in dorsal (top) and palmar (bottom) views.

###

### *TPIT1-492*: A complete, adult intermediate hand phalanx (ray 2-5) (Fig S32).


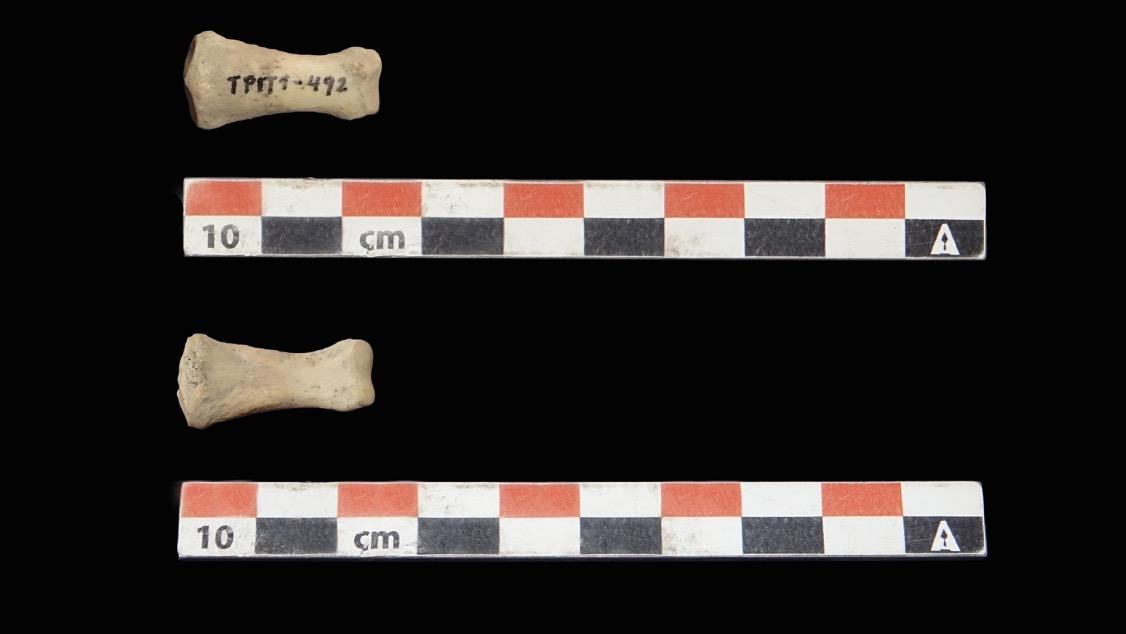


**Fig S32.** TPIT1-492 in dorsal (top) and palmar (bottom) views.

### *TPIT1-495:* A set of distal and intermediate hand phalanges (ray 2-5) (Fig S33). The distal ends of the phalanges are broken off from these specimens.


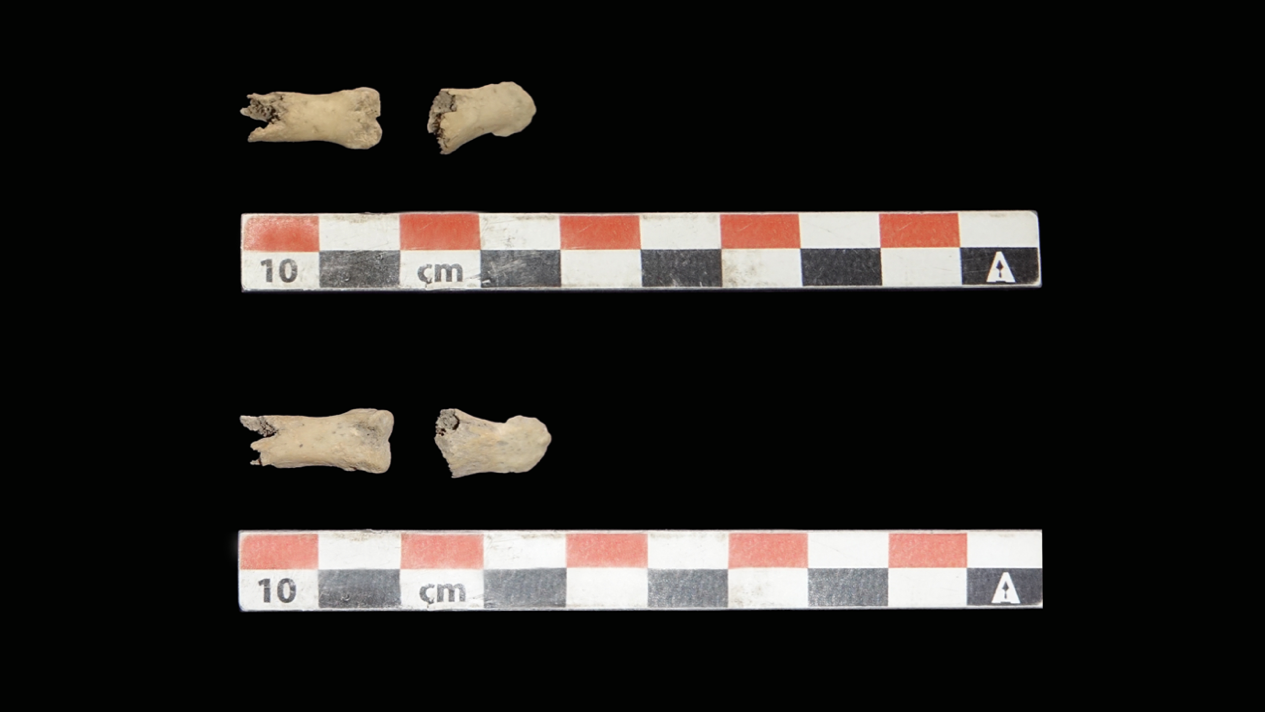


**Fig S33**. TPIT1-495 in dorsal (top) and palmar (bottom) views. The intermediate phalanx is on the left and the distal phalanx is on the right.

### *TPIT1-489:* A proximal hand phalanx (Fig S34). The distal epiphysis is missing from this specimen. There is significant evidence of small carnivore gnawing around the postmortem fracture at the distal portion of the shaft. The proximal epiphysis is minimally exfoliated postmortem.


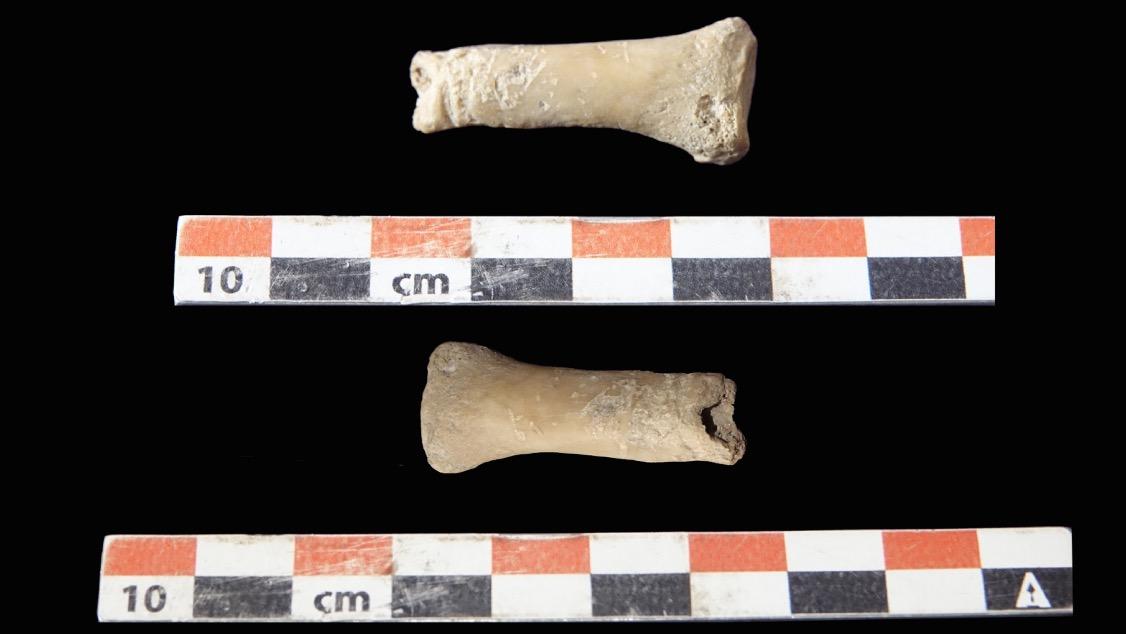


**Fig S34.** TPIT1-489 in lateral (left) and dorsal (right) views.

### *TPIT1-410***:** A complete, adult proximal foot phalanx (ray 2-5) (Fig S35).


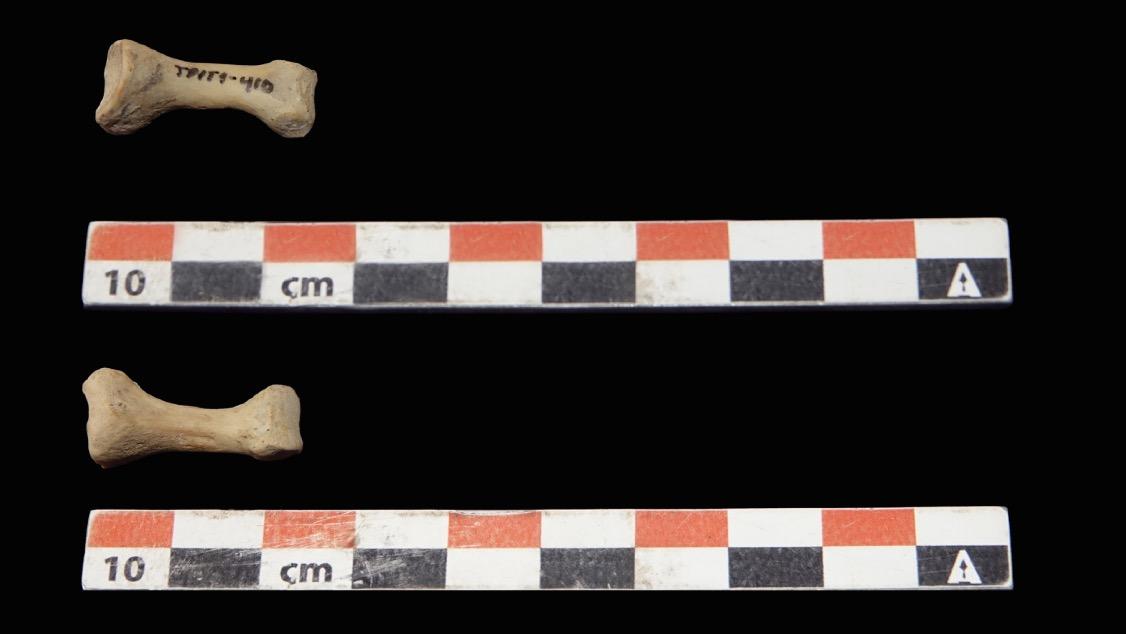


**Fig S35.** TPIT1-410 in dorsal (top) and palmar (bottom) views.

##

### *TPIT1-356:* A right adult cuboid (Fig S36). The bone is well preserved. The height is 34.79 mm, the medial length is 34.73 mm, and the lateral length is 17.14 mm.


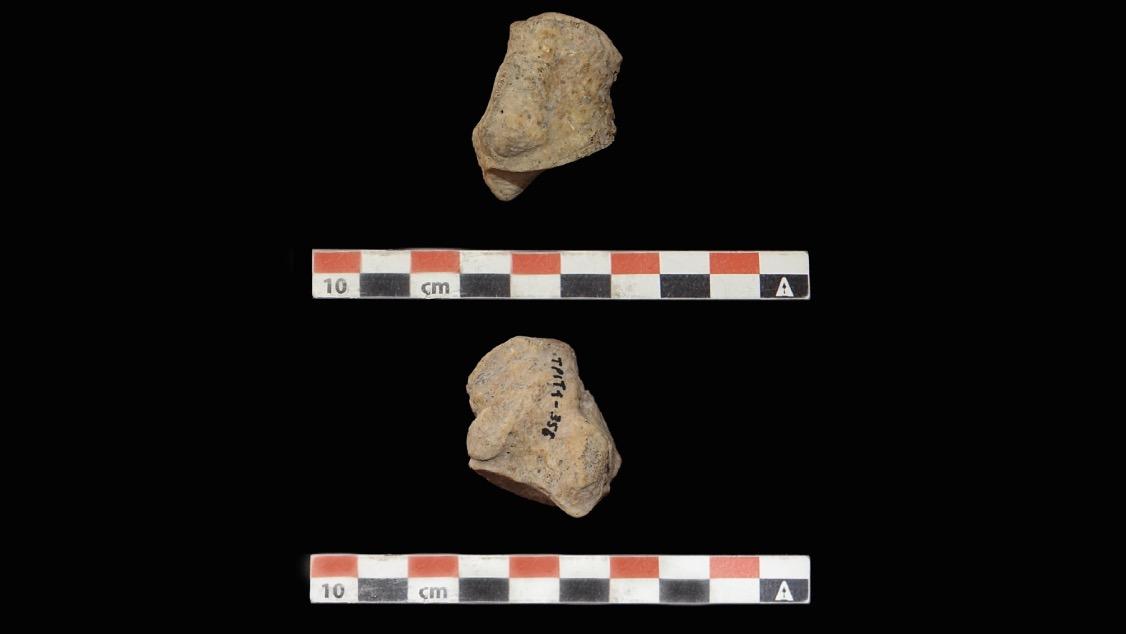


**Fig S36.** TPIT1-356 in dorsal (top) and plantar (bottom) views.

**OTHER**

### *TPIT1-384:* A small long bone, possibly belonging to a perinatal human (Fig S37). Initially, we believed this was the proximal half of a tibia, with a nutrient foramen possibly but the absence of a large nutrient foramen conflicts with this estimate. The current morphology, appreciated both in the photos and in 3D model, cannot reliably be identified as a fetal human bone.


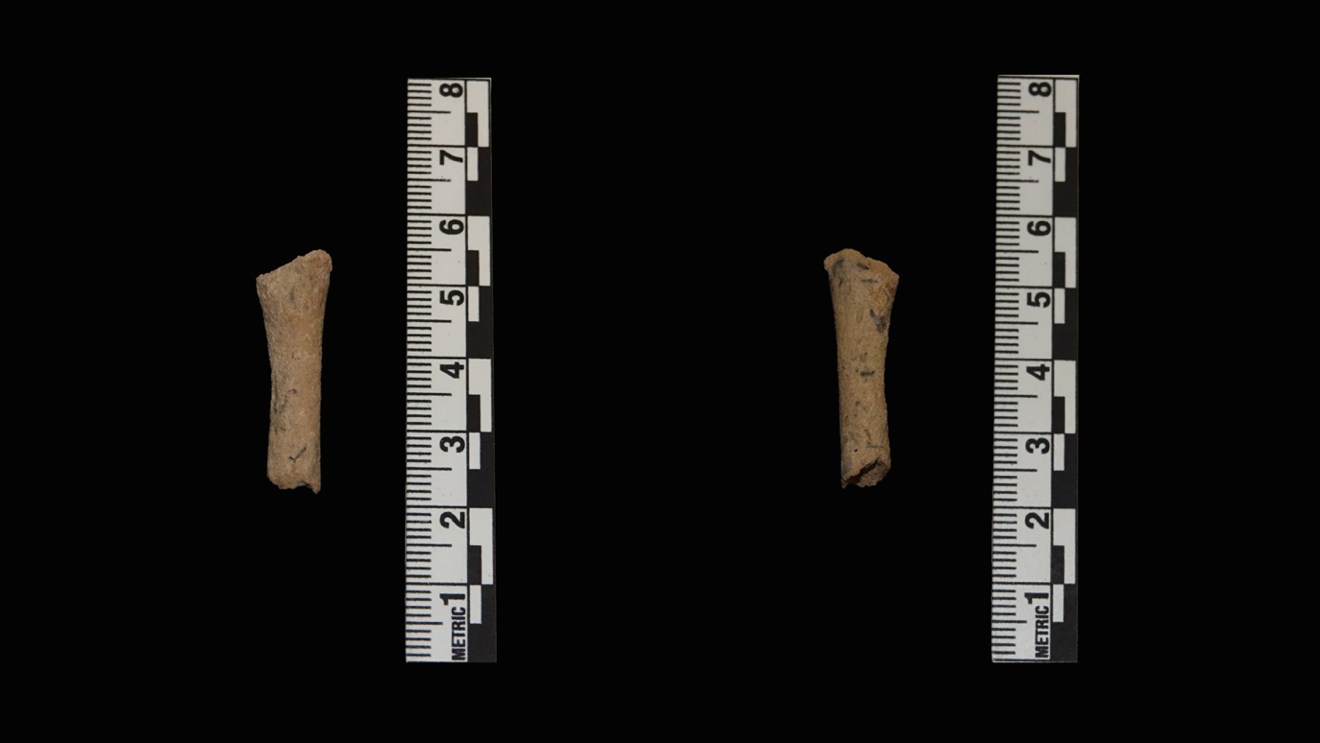


**Fig S37.** TPIT1-384 in anterior (left) and posterior (right) views.

**Fig. S38.** Deamination profile obtained from the read alignment of sample TPIT002 against the human hg19 reference sequence, generated with mapDamage2.0. Cytosine (C) to Thymine (T) changes at 5’ end of fragments are depicted in red, whereas Guanine (G) to Adenine (A) substitutions at the molecule’s 3’ ends are depicted in blue.
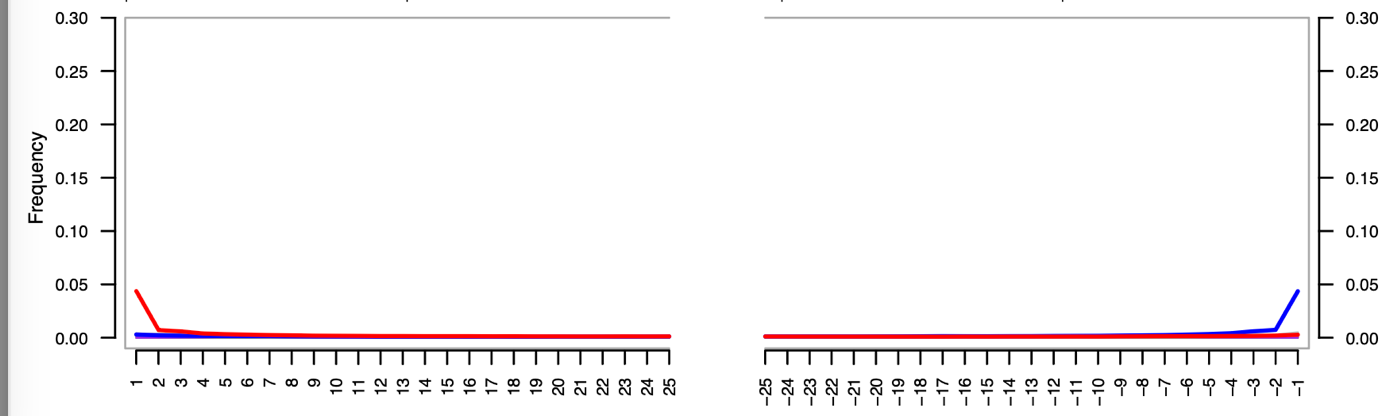


##

## **Supplementary references**

[Baker, B.J., Dupras, T.L., Tocheri, M.W., 2005. The Osteology of Infants and Children. Texas A&M University Press.](https://www.zotero.org/google-docs/?vmZEZJ)

[Buikstra, J.E., Ubelaker, D.H., 1994. Standards for Data Collection from Human Skeletal Remains. Arkansas Archaeological Survey, Research Seminar Series 44.](https://www.zotero.org/google-docs/?vmZEZJ)

[Ferembach, D., Schwindezky, I., Stoukal, M., 1980. Recommendation for Age and Sex Diagnoses of Skeletons. Journal of Human Evolution 9, 517–549.](https://www.zotero.org/google-docs/?vmZEZJ)

[Forseen, S.E., Bruce, C., Gilbert, Patel, S., Ramirez, J., Borden, 2015. Use of the Thoracolumbar Facet Transition as a Method of Identifying the T12 Segment. Journal of Spine 04. doi:10.4172/2165-7939.1000222](https://www.zotero.org/google-docs/?vmZEZJ)

[Rennie, S.R., Eliopoulos, C., Gonzalez, S., 2023. Evaluation of the obturator foramen as a sex assessment trait. Forensic Science, Medicine and Pathology 19, 146–153. doi:10.1007/s12024-022-00514-0](https://www.zotero.org/google-docs/?vmZEZJ)

[Scheuer, L., Black, S., 2000. Skeletal Development and Ageing. In: Scheuer, L., Black, S. (Eds.), Developmental Juvenile Osteology. Academic Press, London, pp. 4–17. doi:10.1016/B978-012624000-9/50003-4](https://www.zotero.org/google-docs/?vmZEZJ)

[Ward, C.V., Latimer, B., 2005. Human evolution and the development of spondylolysis. Spine 30, 1808–1814. doi:10.1097/01.brs.0000174273.85164.67](https://www.zotero.org/google-docs/?vmZEZJ)
