# Supplementary material for: Human occupations at the Alpysbaev Cave (western Tian Shan): Bioarchaeological insights from the Iron Age burial cluster
Source: PLoS One. 2025 Dec 11;20(12):e0333708. doi: 10.1371/journal.pone.0333708 (PMC12697947; doi:10.1371/journal.pone.0333708)
Supplement: Attachment — Submitted filename: Response to reviewers.docx [file pone.0333708.s003.docx]

Dear Dr. Biehl,

Thank you for the opportunity to revise our manuscript. Below, we provide detailed responses to each of the comments, along with a summary of the changes made to the manuscript.

**REVIEWER 1:**

**Thank you for the opportunity to review this manuscript detailing recent findings in a cave site in Kazakhstan. While, I am not an expert on this region of the world - I have worked during this time period and with bioarchaeological remains and the majority of my comments are focused on the skeletal analysis. I found the manuscript and findings very interesting, and certainly worthy of publication; however, there are some areas that need revisions. In summary, I see several areas where additional analyses could be undertaken (mostly in sex estimates) and some updating of the MNI seems necessary. And, more clarity overall in terms of the analysis and DDX is warranted. I outline my comments below by section.**

**Methods**

**For sex estimation - which method(s) were used? Buikstra and Ubelaker is mostly a summary of various methods - so, the authors should specify which methods were used and cite the original articles.**

We agree and have now clarified the specific methods used for sex estimation. We now cite Klales et al. 2012 and Walker 2005 on revised MS lines 118-120.

**Results**

**The clavicles do not represent 3 individuals - they represent at least 2 individuals - since there are two left and one right - it is possible that the right and one of the lefts could be from the same individual - since they are all adult individuals. Unless there is a different reason they are clearly not from the same individual?**

Yes, thank you for this comment. We have revised the discussion of the clavicles and changed the text to indicate that they represented two young adult individuals. Please see revised MS lines 240-246.

**Osteophytes of the vertebrae may also be age-related - this should be discussed as a DDX for the observation as well.**

We have added mention of this to the manuscript on revised MS lines 267-269.

**The rib with the large callus looks like osteomyelitis - this condition can be included as a DDX - particularly with the cloacae.**

We have added mention of osteomyelitis to the manuscript on revised MS lines 318-319.

**Discussion**

**may need to update MNI in this section**

We have updated and clarified the MNI in the discussion sections of the manuscript.

**DJD of the cervical vertebrae is not always associated with use (see comments below and above) - this is also age-related and should be included in the DDX - see:**

**Adams, B. J., Butler, E., Fuehr, S. M., Olivares‐Pérez, F., & Tamayo, A. S. (2024). Radiographic age estimation based on degenerative changes of vertebrae. Journal of Forensic Sciences, 69(2), 391-399.**

See above.

**Pronounced deltoid tuberosity is discussed as being related to male sex and activity - both should be explored together - and not independently. And, may have implications for sex differences in activity - also see note for SI - sex estimation can be made on the humerus.**

We adjusted our discussion of the deltoid tuberosity in revised MS lines 447-451 and no longer link the pronounced tuberosity to a male sex estimate.

**For the rib - I would hesitate to speculate about pain - pain is a very individualized experience and it is not knowable if they were in joint pain - likely pain due to the infection - but it is not knowable how the joints were impacted by this infection or how pain was experienced**

Thank you, we have removed explicit mention of pain.

**Periodontal disease is in the discussion - but, I don't see any discussion of periodontal disease in the results. In fact the results say that no vira were found - and does not clearly state if bacteria were found**

We now explicitly acknowledge that PD was not observed in maxillae or mandibles, but only through the presence of pathogens associated with PD. Please see revised MS lines 479-482.

**Does genetic analysis increase the MNI to 6? Is this due to different genomic signatures? I only saw discussion of sex estimates in the results, so that would only be 2 individuals? Unless these were all from the same tooth? Then, just the duplicating teeth could provide the MNI.**

We do not estimate the MNI based on the produced DNA data. Therefore, the genetic sex estimates do not contribute to the MNI inferred from the other analyses.

**In fact, table 5 should include the tooth for each sex estimate as a column.**

We agree and have added that information to the table.

**Supplemental information**

**The authors use the word vertebrae when referring to a single vertebra - just an editorial comment to change it to vertebra for the singular**

Thank you for catching that spelling error. We have amended all instances of this.

**Also, could the authors use methods of centrum age estimation for these individuals with vertebrae? Such as:**

**Albert, Arlene Midori, and William R. Maples. "Stages of epiphyseal union for thoracic and lumbar vertebral centra as a method of age determination for teenage and young adult skeletons." Journal of forensic sciences 40, no. 4 (1995): 623-633**

**and other related methods?**

We use apophyseal fusion as a means to estimate age in the vertebrae.

**spondylolysis - is related to activity and genetics as well - being well healed is part of spondylolysis - so, this statement can be included with the DDX as part of the evidence for spondylolysis**

Thank you for this comment. We have significantly expanded our discussion on spondylolysis to include explicit mention of its epigenetic nature. Please see revised MS lines 466-478.

**The right humerus could also have a sex estimate following the method of:**

**Rogers, Tracy L. "A visual method of determining the sex of skeletal remains using the distal humerus." Journal of Forensic Sciences 44.1 (1999): 57-60.**

**This could serve as way to verify the observation of robusticity**

**Also, based on the vertical diameter of the humeral head:**

**Stewart has methods for metrics of the humerus, as does:**

**Spradley, M. Katherine, and Richard L. Jantz. "Sex estimation in forensic anthropology: skull versus postcranial elements." Journal of forensic sciences 56, no. 2 (2011): 289-296.**

Thank you for this suggestion, however without a larger sample within which we can seriate, we do not feel comfortable estimating sex from the single humerus.

**Can more detail be given of the perinate tibia? A photo of the nutrient foramen would be helpful. From the photos provided, it does not look like perinate tibia**

We have provided more information in the supplemental text, and now express caution in the perinatal tibia estimate for the very reason you identify. The nutrient foramen is difficult to appreciate in the provided photos, but in the 3D model, the morphology is not entirely consistent with a perinatal tibia.

**Were the discussed cranial fragments not studied? There is discussion of cranial fragments in the text, but they are not in the SI? Nor are they included in table 2 of the main document**

The cranial fragments were not studied as they were not excavated, but we have now amended the text to make this distinction clear. Please see revised MS lines 235-240.
